# Nociceptor subtypes and their incidence in rat lumbar dorsal root ganglia (DRGs): focussing on C-polymodal nociceptors, Aβ-nociceptors, moderate pressure receptors and their receptive field depths

**DOI:** 10.1016/j.cophys.2019.10.005

**Published:** 2019-10

**Authors:** Sally N Lawson, Xin Fang, Laiche Djouhri

**Affiliations:** 1 The Physiology Department, University of Bristol, Bristol BS8 1TD, UK; 2 Qihan BioTech Co. Ltd, Hangzhou, China; 3 Department of Basic Medical Sciences, College of Medicine, QU Health, Qatar University, Doha, Qatar

## Abstract

A recent study with Ca^++^-sensitive-dyes in neurons in whole DRGs (Table 5) found that much lower percentages of nociceptors were polymodal-nociceptors (PMNs) (Emery *et al.*, 2016), than the 50–80% values in many electrophysiological fiber studies.

This conflict highlighted the lack of knowledge about percentages of nociceptor-subtypes in the DRG. This was analysed from intracellularly-recorded neurons in rat lumbar DRGs stimulated from outside the skin. Polymodal nociceptors (PMNs) were 11% of all neurons and 19% of all nociceptors. Most PMNs had C-fibers (CPMNs). Percentages of C-nociceptors that were CPMNs varied with receptive field (RF) depths, whether superficial (∼80%), dermal (25%), deep (0%) or cutaneous (superficial + dermal) (40%). This explains CPMN percentages 40–90%, being highest, in electrophysiological studies using cutaneous nerves, and lowest in studies that also include deep RFs, including ours, and the recent Ca^++^-imaging studies in whole DRGs.

Despite having been originally described in 1967 (Burgess and Perl), both Aβ-nociceptors and Aβ-moderate pressure receptors (MPRs) remain overlooked. Most A-fiber nociceptors in rodents have Aβ-fibers. Of rat lumbar Aβ-nociceptors with superficial RFs, 50% were MPRs with variable medium-low trkA-expression. Despite having conduction velocities at the two extremes for nociceptors, both CPMNs and MPRs have relatively low thresholds, superficial/epidermal RFs and low trkA-expression. For abbreviations used see Table 5.

**Current Opinion in Physiology** 2019, **11**:125–146

This review comes from a themed issue on **Physiology of pain**

Edited by **Lucy F Donaldson** and **Cheryl L Stucky**

For a complete overview see the Issue and the Editorial

Available online 16th October 2019

**https://doi.org/10.1016/j.cophys.2019.10.005**

2468-8673/© 2020 The Authors. Published by Elsevier Ltd. This is an open access article under the CC BY license (http://creativecommons.org/licenses/by/4.0/).

## Introduction

This review firstly examines incidences of nociceptor subtypes in the DRG, especially polymodal nociceptors (PMNs) because knowledge on this was entirely lacking. This lack was highlighted by a discrepancy when a recent study [1^••^] using Ca^++^-sensitive-dyes to track nociceptor activity in neuronal somata in the DRG *in vivo* reported their PMN incidences to be much lower than published electrophysiological studies with values of 40–90% [2]. The Ca^++^-imaging examines neuronal somata in DRGs projecting to all types of tissue, not just skin, as do intracellular DRG recordings, whereas most electrophysiological studies examined PMN incidence in fibers of cutaneous nerves.

A database of >1000 intracellularly-recorded L4/L5 DRG neurons in normal rats recorded at Bristol University (the ‘Bristol database’), was re-examined to determine relative incidences of nociceptor subtypes, after adjustments for known recording bias. The depth (superficial/epidermal, dermal or deep/subcutaneous) of receptive fields (RFs) was critical for understanding this discrepancy. Percentages of the main nociceptor subtypes with RFs at different tissue depths were therefore calculated of contributions of PMNs to L4/L5 DRG neurons, to all nociceptors, and to cutaneous nociceptors. The PMN percentage of all nociceptors was similar to that in the Ca^++^-imaging study [1^••^] while C-fiber PMN percentages of cutaneous C-fiber nociceptors/neurons were consistent with the high percentages from *in vivo* electrophysiological studies of cutaneous nerves. Thus there was no conflict, only different groups of neurons examined in the two types of study. This study should aid future translation between single fiber electrophysiology and whole DRG Ca^++^-imaging studies [1^••^,3^•^,^4^].

The second focus is Aβ-nociceptors, and a subset of these, the Aβ-moderate pressure receptors (MPRs). Their existence is still not universally accepted despite both these groups being first reported in 1967 [8] and despite subsequent careful studies of Aβ-nociceptors in mouse, rat, primate and recently human [5,7, 8, 9] see review Ref. [10^••^].

Because RF depths (superficial/dermal/deep) are related to trkA- and IB4-immuno-intensities in nociceptors, the relationships between nociceptor incidence, subtype, properties, RF-depth and trkA or IB4-binding phenotypes are discussed and a hypothesis to link these is presented.

The review relies on *in vivo* and acutely *in vitro* studies because of the dependence of neuronal phenotypes on the complexity of the *in vivo* internal environment including availability of trophic factors (TFs), which is hard to replicate *in vitro.*

This review is restricted to types and incidences of *normal* nociceptors. It does not address the changes that may occur in nociceptors or silent nociceptors during pain or chronic pain during inflammation, disease or injury.

### Definition of nociceptors

Nociceptors are primary afferent neurons that uniquely signal stimuli intense enough to cause, or potentially cause, damage to the tissues [11] and whose activity usually causes pain. Such stimuli are called **noxious**. Some mechanical nociceptors are high threshold mechanoreceptors (HTMs). Others, such as moderate pressure receptors (MPRs), may have thresholds intermediate between those of HTMs and low threshold mechanoreceptors (LTMRs). Regardless of threshold, both these mechanical nociceptor groups respond better to noxious, than innocuous, stimuli. Unlike LTMRs, they encode stimulus intensity with greater firing through the noxious range and detect sharpness of the mechanical stimulus [12^••^].

In this review the term HTM refers to neurons with a high threshold to noxious mechanical stimuli. Amongst A-fiber nociceptors, HTMs are thus distinct from MPRs (section Moderate pressure receptors). The term C-fiber mechanonociceptors (CM) refers to a C-fiber nociceptor group that responds only to noxious mechanical stimuli.

### Electrophysiological and Ca^++^-imaging approaches

Electrophysiological recordings of sensory fibers *in vivo* determine conduction velocity (CV), firing and sensory properties but not usually soma size. In contrast, fluorescence imaging of Ca^++^-sensitive-dye in DRG neurons *in vivo* can follow activity of multiple neurons simultaneously with their sizes but not CVs [1^••^,3^•^,^4^]. Both approaches may use stimulation (e.g. of the hindpaw) with noxious mechanical and thermal stimuli. Because neuron/fiber classifications rely heavily on CV, the relationship between CV and soma size bears reviewing here.

### Conduction velocity (CV) ranges

C-fiber, Aδ-fiber and Aαβ-fiber CV ranges differ considerably with species, age, gender, temperature, the peripheral nerve or dorsal root used, and, because some CVs slow peripherally, the position along the peripheral nerve [13,14]. These CV ranges therefore need to be determined for every experimental situation. The following are useful methods.

1) Compound action potentials (**CAPs**) (Table 5): Nerve stimulation evokes complex waveforms in the whole nerve, with Aαβ-fiber, Aδ-fiber and C-fiber components [15,16].
2) CV distribution histograms [14] can indicate the boundaries between C-Aδ and Aδ-Aαβ CV ranges in relation to known Aαβ-**LTMR** CVs recorded in that nerve.

**Figure 1:**
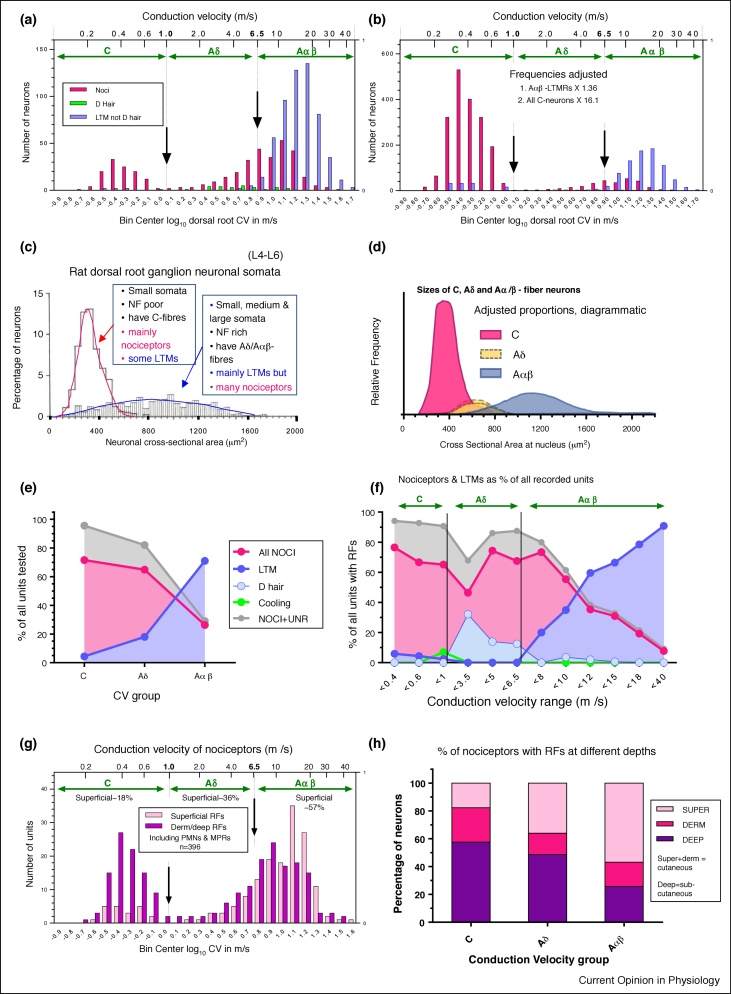
**(a) CVs of nociceptive and LTMR L4/L5 DRG neurons.** Because of the difficulty of showing distributions of C-fiber and A-fiber CVs on the same graph, log_10_ CVs were plotted with bin widths of 0.1 log units; unlogged CVs shown at top of graph. All nociceptors are red; and C-LTMRs and Aαβ-LTMRs (low threshold mechanoreceptors) are blue, and Aδ-LTMRs (D hair units) are green for. Vertical lines and arrows show boundaries between CV groups, determined by compound action potential recordings by X Fang 2002 [17], and confirmed by CV distributions of Aαβ-LTMRs and Aδ-LTMRs (a and b). **(b) Data in (a) adjusted to counter bias during recordings.** Adjustments (Table 1 legend) to compensate for rejection of Aαβ-LTMRs during recording, and to adjust for the underestimated C-neuron population to make up 70% of the total [30], using values shown on graph and Table 1. **(c) The two overlapping size distribution of NF-rich and NF-poor rat L4-L6 DRG neurons**: Size = area = cross-sectional area measured at nuclear, mid-soma, level. NF-poor neurons (open histogram) have C-fibers [21,22]. This population includes mainly nociceptors and unidentified units, including silent-nociceptors, with few LTMRs in these DRGs that project mainly to distal hindlimb. The neurofilament-rich (NF-rich, striped histogram) neurons have A-fibers and are mainly LTMRs, with some nociceptors [21,22]. **(d) Cartoon of area distributions of C, Aδ- and Aαβ-neuronal somata.** Means and ranges for each population from previously published plots [14,21,26], but amplitude of the C-neuron population (red) is adjusted upwards (adjustment 2, see Table 1 legend) to compensate for underrecording of C-neurons. The Aδ-neurons have medium-sized somata that overlap with C-neurons and Aα/β-neurons. It is not known whether the Aδ-neurons are underrepresented (indicated by two dotted outlines). **(e) Percentages of C-, Aδ- and Aα/β-neurons that were nociceptive, LTM or unidentified/silent.** Only Aαβ-percentages were adjusted (Table 1 legend). No C-neuron adjustment needed as comparisons are within CV groups. **(f)** Similar to E but subdivided into smaller CV groups to show the changes in these percentages. **(g) Percentages of nociceptors with superficial RFs.** CVs (log plot) of nociceptors with receptive fields (RFs) that are superficial (red), or in dermal or deeper tissues (pink). The percentage with superficial RFs is low (∼18%) in C-nociceptors, higher in Aδ-nociceptors and increases with CV in Aβ-nociceptors to dominate in the 12–20 m/s range. **(h) Summary graph.** Percentages of C, through Aδ and Aαβ-nociceptors with superficial, dermal or deep RFs are shown.

The frequency distributions of dorsal root CVs of nociceptors and LTMRs in the Bristol data set are shown (log scale) (Figure 1a). The 6.5 m/s borderline between Aδ-Aαβ was determined by the onset of the Aαβ-cutaneous-LTMR distribution (blue) at 6.5 m/s, with most Aδ D-hair CVs being <6.5 m/s (Figure 1a). CAPs (not shown) [17] supported these borderlines. The rat dorsal root A-fiber-nociceptor distribution (red, Figure 1a) is continuous through Aδ-nociceptors and Aαβ-nociceptors as in other species [10^••^], and peaks in the Aαβ-CV range.

#### Low CVs in dorsal roots in the Bristol Data set

The 6.5 m/s Aδ-Aαβ borderline of these Bristol Data is much lower than in many other studies [10^••^]. This is due to the following: 1) Rat age/gender. The CAP peak A-wave CV of sciatic nerve in 300–450d old rats is **1.9** times that of 50-60d rats [18]; our rats were approximately seven week 140–160 g female rats; 2) our CV recordings were at ∼30°C; sciatic nerve conducts ∼**1.24** times faster at 37°C than at 30 °C [18]. 3) CVs were measured along the dorsal root to the soma; rat peripheral nerve A-fibers conduct on average ∼**1.14** times faster than in dorsal root of the same neuron [13]; consistent with this, in peripheral nerve in similar aged rats Aδ fibers conducted up to 8 m/s, and Aαβ-fibers conducted at >14 m/s [14]. A calculation using the above proportions would increase our dorsal root 6.5 m/s Aδ-Aαβ-borderline in older >300gm rats in peripheral nerve to **14** **m/s** (6.5 × 1.9 × 1.14 = 14 m/s) at 30℃, or to **17.4** **m/s** (14 × 1.24 = 17.4 m/s) at 37°C. This illustrates the need to determine CV borderlines on the exact set-up being used for experiment, to ensure CV groups are comparable across methods and species.

### Soma size (area) and fiber CV in DRG neurons

DRG neuronal somata have two overlapping normally-distributed populations of soma areas (cross-sectional area at the nuclear, mid-soma, level) [19,20]. NB These populations are not normally distributed for soma diameter.

The small neuron population has somata that stain poorly for neurofilament (hence NF-poor) and have unmyelinated C-fibers [21]. The neurons in the broad area distribution of mainly larger NF-rich neurons have myelinated A-fibers Figure 1c [21,22].

The NF-poor/small and the NF-rich/mainly large neuron populations exist across many species, but their soma size ranges vary. For example, although NF-poor and NF-rich populations are clear in cat and rat [23, 24, 25], the soma areas are ∼3-fold greater in cat L4 DRG than in rat lumbar DRGs.

## The Bristol dataset

The incidences of sensory neuron subtypes from all intracellular recordings of L4 and L5 DRG neurons in all normal untreated female rats *in vivo*, recorded in the University of Bristol, U.K. by X. Fang and L. Djouhri, were analysed. Other aspects of data from these neurons have been published, but the present analysis of incidence and receptive field (RF) depths is novel. The dorsal root CVs of these neurons and their responses to mechanical and thermal stimuli enabled determination of the sensory type [15], CV (Section: Conduction velocity (CV) ranges) and RF depth (Section: Receptive field (RF) depths of mechanical nociceptors and Supplement 1, Methods). All neurons conducted electrically evoked action potentials from the dorsal root to the soma. For these recordings the DRG and dorsal root were exposed. However, unlike the in vitro skin-nerve preparation, or in vivo preparations for fiber recording, there was no exposure, dissection of, or other interference with, the peripheral nerve distal to the DRG.

### Low threshold mechanoreceptor (LTMR) testing

Mechanical stimuli applied with hand-held stimulators were the primary search stimuli. These were used for RF-type and RF-depth determinations. LTMR subtypes were defined with light touch, brushing, tapping, stretching, light pressure and vibration [15,26,27]. Those that failed to respond were tested with noxious mechanical stimuli.

### Nociceptors

#### Receptive field (RF) depths of mechanical nociceptors

These were defined as follows. Neurons with ***superficial RFs*** responded best to a) gentle needle pressure (not puncture) and very fine pinch of the most superficial tissue with fine, no. 5, forceps. Those with ***dermal RFs*** responded to b) squeeze of a fold of skin tissue including dermis. Those with ***deep RFs*** responded to squeeze across muscles, foot or whole leg with serrated or toothed forceps. Thus the depth of tissue needed to be stimulated mechanically to elicit firing in the soma is referred to as the RF depth. Our interpretation is that ***superficial*** stimulation activates superficial epidermal RFs, ***dermal*** activates RFs or fibers within a fold of dermis, for example, in dermal nerve plexuses, or supplying blood vessels, sweat glands and dermally-projecting lower parts of hair follicles, and ***deep*** relates to RFs/fibers in subcutaneous tissues, for example, deep fascia, nerves, blood vessels, muscle and periostium.

#### RF depths in relation to fiber termination depths

Our recorded RF depths are consistent with knowledge of where different types of nociceptive fibers terminate peripherally. For example, our CPMNs/CMHs have mainly superficial RFs and some dermal RFs. This is consistent with 1. CPMNs being mainly IB4^+^/MrgprD^+^ because IB4^+^/MrgprD^+^ fibers terminate in superficial epidermis (Section: Fiber termination sites) and 2) calculations on the basis of heat responses [28] that monkey C-mechanoheat (CMH) type CPMNs fibers terminate throughout the epidermis and dermis.

Furthermore, most trkA^+^ DRG neurons were CM or AHTM with dermal or deep RFs consistent with studies showing that trkA^+^ and CGRP^+^ DRG neurons project heavily to dermal and deep tissues (Section: Fiber termination sites).

#### Thermal stimuli

Because of the short recording times available for intracellular recordings of C-neurons (sometimes a few minutes) and the complexity of the foot surface, simple thermal stimuli were used. The cooling and cold stimulus was a spray of ethyl chloride. Low threshold cooling units mostly fired spontaneously at room temperature; their firing rate was increased by a brief ethyl chloride spray and reduced by warming. Cold nociceptors were activated by a longer spray of ethyl chloride and noxious mechanical dermal stimuli.

The hot stimulus was 50°C water from a 20 ml syringe ejected onto the skin surface. Because of water flowing away and cooling/evaporation the skin temperature may only have reached 47–49°C; dermal temperatures would be lower. After thermal stimuli, water at room temperature restored normal skin temperature.

The Bristol CMH units responded promptly to a single brief heat, as well as to a noxious mechanical, stimulus.

A-fiber mechanoheat (MH) neurons may be underestimated because a higher rate of heating is required to activate A-nociceptors than C-nociceptors [25], and type I A-fiber nociceptors in monkey have heat thresholds of >53°C [5], higher than our heat stimulus. If this is also the case in rat, our AHTMs may be overestimated.

Percentages of PMNs in our neurons were calculated for comparison with other studies such as [1^••^] in which the hind foot was immersed in 55°C water. Despite their stronger heat stimulus than ours, similar percentages of CMH-PMNs suggest that our stimulus was as effective (Section: MH-type PMNs in Ca^++^ imaging studies).

### Unresponsive neurons

Electrocutaneous stimulation [29] as a search stimulus has the advantage of electrically locating the RF, enabling sensory testing to be limited to that region. It provides good data on incidence of silent/MiHi (mechano-insensitive, heat-insensitive see Section: CMiHi or silent nociceptors, included in unresponsive neurons) fibers in skin (i.e. with superficial or dermal RFs). *In vivo* it would not activate units with deep RFs and possibly only some with dermal RFs depending on electrode location.

For the Bristol Data set, electrocutaneous stimulation was not used. Thus, for each neuron that was not identified as LTMR or nociceptive, complete sensory testing (Section: Nociceptors) in the entire RF of the DRG was needed. This had the disadvantage that strong mechanical testing could move the limb causing the recording to be lost, possibly leading to underestimation of unresponsive neurons. Unlike electrocutaneous stimulation, our method a) provided no RF depth/location information, b) would have included unresponsive neurons with RF depths at all depths but c) may include some L4/L5 DRG neurons with inaccessible RFs on the dorsal surface of the foot, where the midsection of the dorsal surface was glued down for recording stability (Supplementary 1 Methods). Our unresponsive neurons had properties that were very similar to those of well-defined nociceptors (Section: CMiHi, silent or unresponsive-neurons) and therefore do not include LTMRs. Because the leg and majority of the foot including all glabrous surfaces, sides of the foot and toes were accessible, and deep squeezing across leg, foot (medio-laterally) and toes were carried out, the majority of our unresponsive C-neurons are likely equivalent to C-fiber MiHi and silent neurons.

### Dye-injection for immunocytochemistry

After recording was complete, in some identified neurons fluorescent-dye was intracellularly-injected enabling later immunocytochemistry. Relative intensities (percentage of maximum immunointensity compared with neurons in the same section) were calculated [26].

### ANALYSIS 1: Adjustments for bias in C, Aδ and Aαβ sampling

Table 1 shows totals (raw numbers) of neurons in main sensory subdivisions in the >1000 recorded neurons. Adjustments to the Bristol Data to compensate for known bias during recording were made are explained in Table 1 legend. An outline follows.

**Table 1:** Numbers of neurons recorded, adjustments to offset known recording bias and estimated percentages of neuron types after adjustment

	C-neurons				Aδ-neurons			Aαβ-neurons				ALL CVs
	n	n x 16.074	As % of all C	As % of ALL	n	As % of all Aδ	As % of ALL	n	Aαβ	As % of all Aαβ	As % of All DRG	As % of All DRG
Nociceptors	**113**	1816.36	69.3	48.52^1C^	**72**	62.07	1.92^1Aδ^	**211**	**211**	20.95	5.66^1Aαβ^	56.1
LTMRs	**7**	112.52	4.29	3.01	**20**	17.24	0.53	**568**	775	76.96	20.7	24.2
Unresponsive	**38**	610.81	23.3	16.32	**19**	16.38	0.51	**21**	**21**	2.09	0.56	17.4
Cooling	**5**	80.37	3.07	2.15	**5**	4.31	0.13	**0**	**0**	0	0	2.3
Totals	**163**	2620.06	100	70	**116**	100	3.10	**800**	1007	100	26.9	100
All CVs Total n recorded	1079		All Cs	2620			All As	1123		Total Cs+As	3743.3	

Intracellular recordings made by X. Fang and L. Djouhri of neurons in normal (untreated) rat L4 and L5 DRGs *n* = 1079. Nociceptors are included in this table regardless of whether they were thermally tested. Numbers in bold are raw numbers of recorded neurons without adjustment.

There are two main sources of bias for which adjustments were made. Underlined numbers in the Table and below are those after adjustment.

***Adjustment 1) for Rejection of Aαβ-LTMRs during recording*** to avoid their domination of the data set. To determine the extent of this bias, X. Fang carried out a series of experiments accepting all neurons encountered regardless of sensory properties. This determined the unbiased/expected percentage ratio of Aαβ-nociceptors to Aαβ-LTMRs to be 21.4%:78.6%. The number of Aαβ-LTMRs recorded (568, Table 1) was therefore multiplied by 1.364, increasing it to 775, to achieve a ratio of 211:775, equivalent to the unbiased 21.4%:78.6% ratio. This compensated for Aαβ-LTMRs rejected during experiments.

***Adjustment 2) for Underestimation of C-neurons*** due to greater difficulties in recording from them. After making Adjustment 1, the number of recorded C-neurons was adjusted upwards (X16.07) to 2620 to reach 70% of the total neurons, which was the percentage that the small neuron population in rat L5 DRGs contributed to the whole DRG, determined with unbiased counting methods [1^••^]. We assumed that rat L4 and L5 DRGs are similar, as they innervate similar tissues and both run predominantly in the sciatic nerve.

These corrections were applied to Figure 1a data to generate Figure 1b. The total adjusted neuron number including both adjusted A-neurons and C-neurons is 3743 (Table 1).

Superscripts C, Aδ and Aαβ refer to neurons with CVs in these ranges. Numerals 1-3 refer to the order in which values are used in examples of calculations to determine totals of a nociceptor subtype, in this case polymodal nociceptors (PMNs), (see Table 3, and Legend) and for generating other percentages used in Figure 3. 1 or 2 decimal places are used to enable easier tracking of calculations.

#### Adjustment 1 to Aαβ-LTMRs

Some of these were rejected during recording. This rejection was to avoid them swamping the data set. To offset this loss, Aαβ-LTMRs (Table 1) were multiplied by 1.364 from 568 to reach 775. All other neuron types were accepted during recording.

#### Adjustment 2 to C-neurons

C-neurons were underestimated due to small soma size (fewer penetrations) and apparent fragility. After Adjustment 1, the number of recorded C-neurons was adjusted upwards (X16.2) to contribute 70% to the neuron total because this is the unbiased percentage that the small (type B) neuron population (thus C-neurons see Section: Soma size (area) and fiber CV in DRG neurons) contributes to all rat L5 DRG neurons [30].

Figure 1b shows the effect of these adjustments on the raw data in Figure 1a, note the greater similarity in proportions of C-neurons and A-neurons to those of cell areas from whole tissue sections in Figure 1c. Figure 3 shows Pie Charts of the results of these plus later calculations.

#### Possible bias without adjustment

The percentage of Aδ-neurons is small (Figure 3). However, if their smaller sizes caused Aδ-neurons to be significantly underestimated relative to Aβ-neurons, a peak at around 600–800 μm^2^ would be expected in the normal distribution of NF-rich neurons sizes, but this is not seen (Figure 1c), suggesting that any error is not great. No adjustment was made, since we had no objective basis for this.

#### Consistency of data and calculations

**Figure 2:**
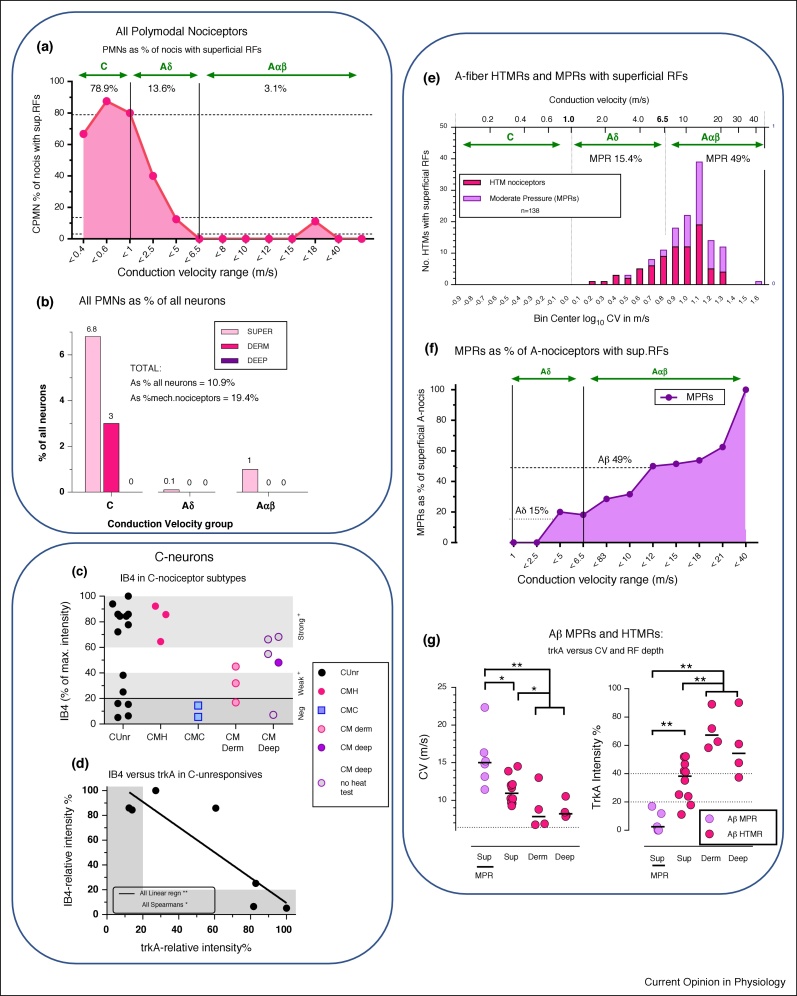
**(a) Percentages of nociceptors with superficial RFs that are PMNs.** This is high (79%) for C-nociceptors, decreasing through Aδ (average ∼14%) to almost none (∼3%) of Aαβ-nociceptors. **(b) Percentage contribution of PMNs to all neurons.** The vast majority of PMNs were C-nociceptors with superficial RFs. None with deep RFs and no A-nociceptors with dermal RFs showed responses to noxious heat. **(c) IB4 relative immuno-intensities relative to C-nociceptor subtypes.** This includes only units in this Bristol data set. It is a different plot of data most of which were previously published [26]. Here, the limits are defined more precisely, in that deep HTMRs and unresponsive units are included only if comprehensively tested with all types of noxious mechanical and noxious thermal stimuli, except for open symbols in CM deep that were not heat tested. The grey bands of 0–20% indicate negativity for IB4, 20–40% indicates weak staining, and 60–100% indicates strong staining. Note the gap in the CUnr (C-unresponsive) units between 40–70% suggesting two possible groups, strongly IB4^+^ and negative or weakly IB4^+^. Also note the IB4^+^ CMH and IB4^−^ CMC units. Dermal and deep HTMRs were mostly negative to medium but not very strong IB4-immunointensities. **(d) trkA versus IB4-immunointensities in C-Unresponsive neurons**: All these neurons are from this Bristol database, and each had immunocytochemistry for trkA and IB4 carried out on different sections of the same neuron. **(e–g) A-fiber HTMRs and MPRs.** **(e) Incidence of A-fiber HTMRs and MPRs with superficial RFs.** For definitions see Sections: A-fiber PMNs and Moderate pressure receptors. MPRs are nociceptors that fire in response to moderate, non-noxious, pressure, but fire faster to higher intensity mechanical stimuli, encoding intensity through the noxious range [12^••^]. High threshold mechanoreceptors (HTMRs) are nociceptors with higher mechanical thresholds than MPRs that do not respond, or respond poorly, to moderate pressure. **(f) MPRs as a percentage of A-nociceptors with superficial RFs.** This increases with CV, being low (15%) in the Aδ-range, and rising to 49% in the Aαβ-range especially above 16 m/s (log 1.2) when the incidence is declining. NB All MPRs had superficial RFs. **(g) Aβ-MPRs and HTMRs: trkA versus CV and RF depth.** Redrawn from Fang *et al.* [9]. In Aαβ-nociceptors: MPRs have the fastest CVs and lowest trkA-expression. trkA-expression was highest in nociceptors with dermal or deep RFs, was next highest in nociceptors with superficial RFs and lowest in MPRs.

To establish whether data of the two experimenters were consistent, their data were plotted separately for graphs in Figures 1e,h, 2 a and d. All clearly showed the same patterns for both experimenters.

### Areas of neurons with C-fibers, Aδ-fibers and Aαβ-fibers

Figure 1d is a cartoon of area distributions of neurons with C-CVs, Aδ-CVs or Aαβ-CVs, from our published size ranges and means [14,21,26], with amplitudes adjusted to compensate for C-neuron underestimation (Section: Adjustment 2 to C-neurons, Table 1 legend). The C-neuron and A-neuron area distributions are normally-distributed, with few C-neurons of larger sizes. Aδ-neurons are NF-rich with intermediate-sized somata that partially overlap both C-neuron and Aαβ-neuron distributions [14,21,22,26].

A demonstration of this area distribution/CV relationship is clear in Figure 3 of Chisholm *et al.* [3^•^]. This shows neurons activated (increased intrasomal Ca^++^ levels) by high-intensity ‘C-fiber’ but not low-intensity electrical stimulation, suggesting that they were nociceptive. Size distributions of neurons selectively-activated by noxious ‘C-fiber’ stimuli suggest (Figure 1d) that many were A-fiber nociceptors. The term ‘*Noxious*’ rather than *‘C-fiber’* stimuli would therefore seem more appropriate. Since NF-poor C-neuron areas are normally distributed (Figure 1c) the two sides of the distribution are mirror images, and the upper end of the C-neuron distribution [3^•^] is probably ∼560 μm^2^. Subtraction of this distribution from all neurons activated selectively by noxious stimuli could show the extent of A-nociceptors. Comparison with Figure 1d suggests that these include Aδ-nociceptors and Aβ-nociceptors, illustrating the usefulness of these soma area distributions [3^•^].

**Figure 3:**
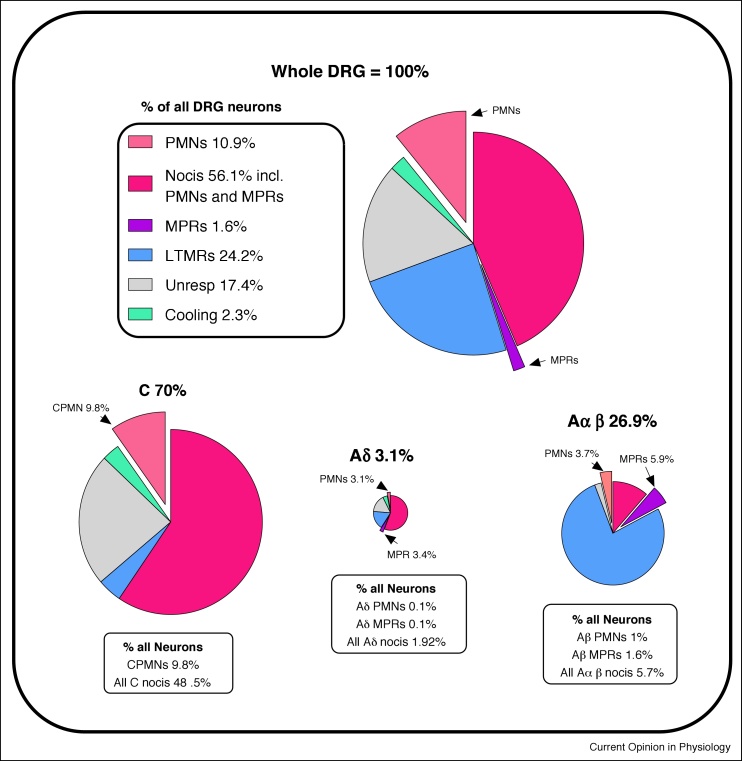
**Pie charts of neuronal subtypes.** Percentage contributions of CV groups and main neuronal types to the rat L4/L5 DRG. The top chart is all neurons, and below the C, Aδ and Aαβ charts are shown with their sizes approximately proportional to their contributions to the total population, and the percentage contributions of PMNs and MPRs to the DRGs. Arrows and percentages at C, Aδ and Aαβ-charts show contributions of PMN and MPR neurons to the total in that CV group.

### Analysis 2: C, Aδ and Aαβ-nociceptor incidence

The generally accepted narrative remains that C-neurons and Aδ-neurons are nociceptive and that Aαβ-neurons are all LTMRs. However, this is not the case, as C-LTMRs, Aδ-LTMRs (D hair units) and Aαβ-nociceptors are present across many species [10^••^,^23^], see Figure 1a-b, e and f.

The following calculations in this section were made on the Bristol Data set after the adjustments (Table 1, Section: ANALYSIS 1: Adjustments for bias in C, Aδ and Aαβ sampling). The percentage of *all* neurons that were nociceptive was highest (69%) in the C-range declining to ∼21% in the Aαβ-range, with the opposite trend for LTMRs (Figure 1e, Table 1). Looking at smaller CV subdivisions (Figure 1f), percentages of nociceptors remained high from C to low Aβ-CVs, except for an Aδ dip coinciding with the D-hair peak. In the Aβ-CV range from >8 m/s, the nociceptor percentages declined progressively as CV increased due to the increasing percentage of Aαβ-LTMRs. The highest percentages of Aαβ-LTMRs for CVs >20 m/s (Figure 1f), coincide with their declining incidence, (Figure 1b). For contributions of the major subtypes to the whole DRG and to the different CV groups see Pie Charts (Figure 3).

### Incidences at different receptive field (RF) depths

Percentages of mechanical nociceptors with RFs at different depths (Section: Receptive field (RF) depths of mechanical nociceptors and Supplement 1) differed markedly between C-nociceptors and A-nociceptors. Only ∼18% C-nociceptor RFs were superficial (Figure 1g-h, Table 2a ) suggesting that most C-nociceptors monitor dermal and deeper tissues. Of Aβ-nociceptors, ∼57% were superficial, more than Aδ-nociceptor or C-nociceptor RFs (Figure 1g and h, Table 2a) consistent with the importance of rapid conduction in nociceptors detecting external threats. C-nociceptors dominate the innervation of deep tissues, and Aβ-nociceptors dominate the cutaneous innervation.

**Table 2:** Percentages of all nociceptors with RFs at different tissue depths and of PMNs at different depths

**2a**			
	% of mechanical nociceptors with RFs at different depths		
	C	Aδ	Aβ
Superficial	17.7^2C^	36.1^2Aδ^	56.9^2Aβ^
Dermal	24.8	15.3	17.53
Deep	57.5	48.63	25.6

**2b**			
	% mechanical nociceptive RFs at different depths that are PMN/MH + MHC/MC		
	C	Aδ	Aβ
Superficial	79/57.9/23.5^3C^	13.6/13.6/0^3Aδ^	3.1/2.1/1.0^3Aβ^
Dermal	25/8.3/13	0*/0*/0*	0/0/0
Deep	0/0/0	0*/0*/0*	0/0/0

The depths of nociceptive RFs were defined on the basis of noxious mechanical stimuli as follows: those with superficial RFs responded to needle pressure (not puncture) and fine pinch of superficial tissues with fine forceps, those with dermal RFs responded only to squeeze of a fold of skin tissue including dermis, and those with deep RFs responded only to pressure to tissues including deep fascia, muscle, and periosteum by squeezing across muscles, the foot or whole leg.

*indicates only 1-3 neurons tested with noxious thermal stimuli, so less weight should be placed on these calculations.

Table 2a Percentages of all nociceptors with RFs at different tissue depths regardless of whether they were tested with noxious thermal stimuli as for Table 1.

Table 2b Percentages of nociceptors with RFs that were PMN/MH + MHC/MC at the different tissue depths. MH + MC includes all such units of the PMN group. All nociceptors included had been tested for noxious mechanical and noxious heat, but not all were tested with noxious cold, thus percentages from which MC and MHC were calculated were smaller than numbers than for MH. Nociceptors included high-threshold mechanoreceptors (HTMs), and polymodal nociceptors (PMNs), either mechano-heat units (MH), mechano-cold (MC) or both (MHC). This data set had no neurons that responded only to noxious heat. PMNs include MH, MC and MHC. Non-noxious cooling-sensitive C-neurons (often showing ongoing firing) are not considered nociceptive, and are thus excluded from this Table, but included in Table 1, Figure 1f and Figure 3.

Superscripts as in Table 1; for example, ^2C^ and ^3C^ are the second and third numbers used to calculate C-fiber PMN contribution to the total number of DRG neurons. Note that ^3C^ refers to 3 numbers 80/55/25. These are the percentages of C-nociceptors with superficial RFs that were PMNs/MH + MHC/MC respectively. These calculations were repeated for these three types of unit, and then for the different tissue depths (superficial, dermal or deep) of receptive fields.

## C-fiber nociceptor subtypes

It is not possible to cover these types in great depth. The main focus is to define the groups in order to calculate their incidence and to summarise aspects of their functions.

### C-mechanically-sensitive afferents (CMIAs)

CMIAs include C-polymodals **(CPMNs)** and C-mechanonociceptors **(CMs). CPMNs** include C mechano-heat units **(CMHs**), C mechano-cold units (**CMC**s) and C mechano-heat + cold units (**CMHCs**). CMs are sometimes called C high threshold mechanoreceptors (**CHTMs**).

#### C polymodal nociceptors (CPMNs)

The term polymodal nociceptor (PMN) was originally defined [31,32] as C-fiber nociceptors with superficial cutaneous receptive fields, that respond to more than one type of noxious stimulus applied to the skin, usually mechanical plus either noxious heat or noxious cold or all three, that is, CMH, CMC and/or CMHC. Their mechanical thresholds were ‘elevated but not extreme’ in cat [31,32]. In human hairy skin these thresholds were 7–90 mN, mostly below 35 mN and in rat they ranged from 0.5–40 mN [33]. Thus, they have a wide range of mechanical thresholds, starting relatively low. Heat thresholds in human microneurography were lower in CMHs (40.7℃) than in CH units (48℃) [34^••^]. Thus, CMH units tend to have relatively low thresholds. This may be related to their very superficial receptive fields (Figure 2a, Table 2b and analysis, Sections: PMNs as a percentage of all DRG neurons, Fiber termination sites).

Despite the specificity of the original term (above), in this review, the term PMN is used for all Bristol nociceptors that responded to mechanical and thermal noxious stimuli, whether MH, MHC or MC, and regardless of CV-range or RF-depth. These subdivisions are detailed inTable 2b and totals are shown in Figure 2b. This inclusive use of the term PMN is for comparison with studies that cannot differentiate between CVs or RF depths [1^••^,3^•^,^4^]. However, the functional specificity of the original definition (above) is useful where subdivisions of CV and RF depth are available and is therefore also made clear throughout.

CPMN incidence is calculated in Section: Analysis 3: incidence of CMIAS: CPMNs, CMHs and CMs.

##### C mechanoheat (CMH) units

These were the dominant type of CPMNs in the Bristol database and most other studies. Calculations of incidence are included in Section: Analysis 3: incidence of CMIAS: CPMNs, CMHs and CMs.

##### C mechanocold (CMC) units

CMCs were infrequent, had high mechanical thresholds [35] and responded to noxious cooling. Their incidence in the Bristol Data is found by subtracting CMH + CMHC (Table 3b ) from CPMNs (Table 3a) see Section: Analysis 3: incidence of CMIAS: CPMNs, CMHs and CMs.

**Table 3:** Percentage contributions of PMN and MH only units to the total DRG neuron population

**3a**	% contribution of PMNs to the DRG			
	C	Aδ	Aβ	ALL
Superficial	6.78	0.1	0.99	**7.87**
Dermal	3	0*	0	**3**
Deep	0	0*	0	**0**
Totals	**9.78**	**0.1**	**0.99**	**10.87**

**3b**	% contribution of MH + MHC units to the DRG			
	C	Aδ	Aβ	ALL
Superficial	5	0.1	0.66	**5.76**
Dermal	1	0*	0	**1.0**
Deep	0	0*	0	**0**
Totals	**6.0**	**0.1**	**0.66**	**6.76**

Table 3a To obtain estimates of the numbers of all PMNs or of the MH or MC units, for each depth, percentages from Table 1, Table 2 were converted to proportions and appropriate proportions were multiplied together resulting in Table 3a. For example, to obtain the contribution of CPMNs with superficial RFs to all DRG neurons, the following proportions are multiplied together: that of all DRG neurons that are C-nociceptors (1C/100 = 0.485), that of all C-nociceptors with superficial RFs (2C/100 = 0.177) and that of C-nociceptors with superficial RFs that are PMNs (3C/100 = 0.79). The product is multiplied by 100 to obtain the estimated percentage of all DRG neurons that are CPMNs with superficial RFs, that is, **6.8**%.

Repeating this for units with dermal and deep RFs results in the total percentage contribution of all CPMNs to all L4/L5 DRG neurons, that is, **9.8**%. Repeating these calculations for Aδ-PMNs and Aαβ-PMNs with RFs at these different depths and adding these to the CPMN total provides the cumulative percentage estimate of all PMNs to the whole DRG neuron population of **10.87**% (Table 3a).

Table 3b repeats the above for heat-responsive nociceptors only (PMNs that are MH or MHC, but excluding MC). This is a smaller contribution, **6.8%,** of all PMNs to all DRG neurons.

Thus, percentages for all CPMNs, just CMH + CMHC units and for just CMC units only are 9.8%, 6.8% and 3% (9.8 minus 6.8) respectively. Because more units were tested for noxious heat than noxious cold, the CMH + CMHC unit estimations of **∼7**% are more reliable than the CPMNs, for which a range of **9-13**% is a reasonable estimated range for all PMNs.

*indicates only 1-3 neurons tested with noxious thermal stimuli, and that had clear RF depth recorded, so less weight should be placed on these dermal and deep RF Aδ calculations.

#### CMs or CHTMs

CMs respond to noxious mechanical, but not noxious thermal stimulation. From reciprocals of the first numbers in Table 2b), 21%, 75% and 100% of C-nociceptors with superficial, dermal and deep RFs respectively were CMs. CMs have been reported as having mechanical thresholds that were higher [31,32] in cat than CMHs or similar [29] in humans to CMHs. In the Bristol data (rat) they had higher mechanical thresholds than CMH units, at least partly due to most having deeper RFs; most required squeeze of a skin fold or deep tissues with toothed forceps or serrated flat forceps for maximum firing. Primate cutaneous CMs had much higher transcutaneous electrical thresholds than CMHs [34^••^]. Again, this is likely to be due to deeper RFs than CPMNs and/or different ion channel expression/activation.

### C-fiber mechanically insensitive afferents (CMIAs)

There are two subgroups, C-Mechano-insensitive Heat-insensitive **(CMiHis)** afferents and C heat nociceptors **(CH)s**. CMIAs showed much greater activity dependent slowing (ADS) with microneurography in primate than other C neuron groups including CPMNs and C-LTMs [34^••^], suggesting difference/s in ion channel expression or activity.

Growing evidence implicates CMIAs in chronic pain. In primate, CMIAs were initially silent or had extremely high thresholds and were more highly activated by inflammatory mediators than CPMNs [36]. They respond to capsaicin and histamine but are not β-alanine-sensitive [37], suggesting they are not MrgprD^+^/IB4^+^ (Section: Transduction and CPMNs: a role for keratinocytes?). Also, because in human the firing of CMIAs, but not CPMNs increased during sustained mechanical stimuli, CMIAs were proposed to contribute to the increasing pain experienced with constant mechanical pressure [38].

#### CMiHi or silent nociceptors, included in unresponsive neurons

These have been called silent nociceptors and more recently CMiHi [29]. CMiHi neurons probably make up the majority of our unresponsive neurons (Section: Unresponsive neurons).

Functionally, microneurography in human patients with neuropathic pain and fibromyalgia showed that it was the CMiHi/silent nociceptors that showed spontaneous firing [39]. These are therefore likely to be an important cause of spontaneous ongoing pain [40].

#### C heat nociceptors (CHs)

These were not observed in the Bristol Database although they have been reported in rat (3–4% of nociceptors) [41,42]. Their heat thresholds are higher than those in CPMNs in humans [34^••^]. If they also have higher heat thresholds than CPMNs in rat, our heat stimulus may not have activated them (Section: Thermal stimuli). In this case they may be included in our C-unresponsive neuron group, increasing their percentage but not affecting percentages of other neurons.

### C-cooling receptors

In the Bristol data this term means responsive to non-noxious cooling. These were a small population that showed ongoing firing at room temperature and fired faster after a brief ethyl chloride spray. They are shown in green (Figures 1f and 3).

## CVs of C-nociceptor subtypes

**CVs:** The slowest CVs were of CHs (C-heat nociceptors) in mouse, which conducted slower than other C-neurons [43]. In humans, CVs of CMIAs (CH and CMiHi) were slower than CMH units [29,34^••^]. The fastest CVs in the Bristol data were C-cooling units which were faster (median 0.7 m/s) than CMH (0.54 m/s) and C-unresponsive neurons (0.45 m/s). Between these extremes are CMHs in human which conducted faster than CM and faster than CMIAs [29]. CVs of CMHs in mouse, unlike human, were similar to CMs, and similar to human they were faster than CHs [43].

## Analysis 3: incidence of CMIAS: CPMNs, CMHs and CMs

These were determined using the Bristol database as described in Table 2, Table 3, Table 4 and their Legends. Percentages are of all neurons (Table 1) or of mechanical nociceptors because we saw no heat-only or cold-only nociceptors.

**Table 4:** Percentage contributions of CPMN and CMH-type CPMNs only to all mechanical nociceptors, C-nociceptors, cutaneous C-nociceptors, and C-nociceptors with superficial epidermal receptive fields

	% contribution of CPMNs or CMH + CMHC to :				Superficial epidermal C nocis
	All neurons	All nociceptors	C-nociceptors	Cutaneous C-Nocis	
CPMN	9.8	17.4	20.2	47.5	79
CMH + CMHC	6	11	12.1	29	58

Note that all CPMNs and CHs are cutaneous, since no CPMNs or MHs had deep receptive fields.

CPMNs, including CMCs, and CMHs (CMH + CMHC) excluding CMCs were both calculated as a percentage of:-

**All nociceptors** using a simple conversion since 56.1% of all neurons were nociceptive, Table 1. Table 3a,b values x 100/56.1, that is,×1.78, provides the values. CPMNs including CMCs contributed 9.8 × 1.78 = **17.44%** to all nociceptors while CMH + CMHC contributed 6 × 1.78 = **12%**. All PMNs including C, Aδ and Aβ PMNs and MHs contribute **19.4%** to the total nociceptive population, while MH + MHC units of all CV groups contribute **12.06%.**

**All C-fiber nociceptors**. C-fiber nociceptors were 48.52% of all neurons (Table 1) so values in Tables 3 were multiplied by 100/48.52 = 2.06. CPMNs were 9.8 × 2.06 = **20.2%** and CMH + CMHC contributed 6 × 2.06 = **12.1%**.

**Cutaneous C-nociceptors** were calculated for comparison with cutaneous nerve-based electrophysiological studies. Starting with the percentage of C-nociceptors that were CPMNs (above, 20.2%) times 100 divided by the percentage of all C-nociceptors that had superficial + dermal RFs from Table 2 that is, 17.7 + 24.8 = 42.5%. Thus 20.2x (100/42.5) = **47.5% for all CPMNs** or 12.1x(100/42.5) = **29.4% for CMH + CMHC,** each as a percentage of cutaneous (superficial + dermal) C-fiber nociceptors.

**Superficial Epidermal C-nociceptors:** percentages directly from Table 2b, Row 1; **79%** for CPMNs and **58%** for CMH + CMHC units.

Aδ-PMN and Aβ-PMN and MH units can also be calculated as above.

### PMNs as percentages of nociceptors with RFs at different depths

The percentage of C, Aδ or Aβ-nociceptors with superficial RFs that were PMNs was much greater for CPMNs (79%), than for A-fiber PMNs (Aδ-PMNs 13.6%, Aβ-PMNs 3.1%) Figure 2a, Table 2b. Values for CPMNs as a percentage of C-nociceptors with dermal and deep RFs were 25% and 0% respectively, Table 2b. The few A-PMNs in the Bristol data set had superficial RFs (Figure 2b) but note that few Aδ dermal/deep units were thermally tested (Table 2b asterisks) making these Aδ values less reliable.

### PMNs as a percentage of all DRG neurons

The contribution of PMNs with all RF depths and in all CV ranges were calculated (Figure 2b, Table 2). Of all DRG neurons, 10.9% were PMNs; most of these, 9.8% of all neurons, had C-fibers (Figure 2b, Table 3a). A total of **6.8**% of all DRG neurons were MH-responding or MHC-responding units, of which most (**6%** of all neurons) had C-fibers (Table 3b). Figure 2b shows the dominant contribution of the CPMNs with superficial and dermal RFs to the entire PMN population in the DRG. Pie charts (Figure 3) show the contributions of CPMNs, APMNs and other neuronal types to the whole DRG and to the C, Aδ and Aαβ-neurons.

### CPMNs and CMHs as percentages of different neuronal groups

For the purposes of comparisons with different methods of study, for example, fluorescence *in vivo* Ca^++^ imaging studies and electrophysiological fiber studies, the percentage contributions from Bristol Data of CPMNs and CMH-type CPMNs (CMHs + CMHCs) to the following are provided in Table 4: all DRG neurons, all nociceptors, C-nociceptors, cutaneous C-nociceptors and cutaneous C-nociceptors with superficial RFs. These values vary from 9.8–79% for CPMNs and 6–58% for CMH + CMHC units, the lowest values being of all DRG neurons, and the highest for superficial epidermal RFs, up to 79% for CPMNs and 58% for CMHs + CMHCs (Table 4). For all cutaneous nociceptors including superficial and dermal RFs, the calculated values are 47.5% for CPMNs and 29% for CMH + CMHC units. It is therefore important to define which group of neurons is the 100% to which the percentage relates, especially when comparing different methods.

### CPMN/CMH percentages in electrophysiological studies

We examine the reported variability of CPMN incidences listed in [2] from 11% to 100% in 11 electrophysiological studies. Most (8/11) of the percentages were of a total (100%) of either responsive (i.e. non-silent) cutaneous C-fibers or of cutaneous C-nociceptive fibers being CPMN or CMH, from *in vivo* experiments on cutaneous nerves. Their values of 65–86% are within, or slightly higher, than the calculated ranges on Table 4 with some being closest to the higher percentage calculated for superficial RFs (79% for CPMNs, and 58% for MHs). This is consistent with greater proportions of superficial versus dermal RFs being activated by primary search stimuli, such as transcutaneous stimulation [38], Von Frey hairs, fine pinch or needle. Whether the 100% for each was of all responsive cutaneous C-fibers or only cutaneous C-nociceptors has a small effect, because the vast majority were mechanical nociceptors, but whether they included MH only or also MC units would have a greater effect. Two studies were on the skin-nerve preparation. One had a similar value to the above (73% of cutaneous C-nociceptors were CMHs) [44]. The other had a lower value of 41% for CMH of C-nociceptors [45], perhaps because the heat was applied to the dermal surface, and most CMH RFs are in the stratum granulosum of the epidermis (Table 2b, Figure 2a, Section: Fiber termination sites). The lowest value (11%, from our paper) [46] relates to CMH units in all C-nociceptors in the DRG including dermal RFs (not limited by using a cutaneous nerve); this is also calculated here to be 11% (Table 4). Thus a value of 11% of CMH in all C-nociceptors including all RF depths (when recording from the whole DRG) is entirely compatible with a value of 70–80% of cutaneous C-nociceptors being CMHs, especially if the stimulus favours epidermal RFs.

### MH-type PMNs in Ca^++^ imaging studies

In the Emery paper [1^••^] the percentage for MH-type PMNs was only 6.7%; however subsequent additional data using the same methods raised this value to ∼**13.5%,** not significantly greater than the 6.7% (personal communication from D.I MacDonald, E.C. Emery and J.N. Wood). It is important to understand the extent of deep tissue stimulated. Emery *et al.* [1^••^] used *‘mediolateral pinch with serrated forceps in the middle of the glabrous skin, that would likely have activated skin and subdermal tissues such as deep fascia, small muscles and deeper blood vessels, but not bone’* (Personal communication, Emery and Wood). The closest calculation on the Bristol Data was therefore MHs (all CVs) as a percentage of all nociceptors (all CVs). All MHs as a percentage of all neurons = 6.8% (Table 3a). Since 56.1% of all DRG neurons are nociceptive, the total is (6.76/56.1) × 100 = **12%** of all nociceptors. The CMHs contribute 11% (Table 4) of these and AMHs 1%. This value is close to the **13.5%** reported above and see [1^••^]. The extent of the deep tissue stimulated is important; the more deep RFs included, the lower the percentage of PMNs/MHs because deep RFs are not stimulated by heat from outside intact skin.

Another paper also using Ca^++^ imaging methods [4] reported very high values of response to more than one stimulus. For comparison with preceding calculations, we excluded neurons responding to non-noxious stimuli (brush and 20℃ stimulation) since these do not contribute to nociceptor polymodality in its original sense of noxious stimuli (Section C polymodal nociceptors (CPMNs)). We calculated their MH-PMN percentage from their Figure 4F pinch and noxious heat data. The percentage of mechanical nociceptors (pinch) responding to noxious heat (comparable to our Bristol data) was 26/72 = **36%**. It is not clear how much subcutaneous tissue was included in the ‘noxious pinching with serrated forceps’. Their value is higher than ours for all nociceptors including those with deep RFs (12%, previous paragraph) and with no subcutaneous tissue is calculated to be 29% for CMH (Table 4) and ∼**32%** for MHs with all CVs. Their slightly higher value suggests little subcutaneous tissue is included, and/or that the pinch being ‘applied multiple times’ could cause sensitisation and increase the number of fibers responding as MH units [1^••^,^2^].

**Figure 4:**
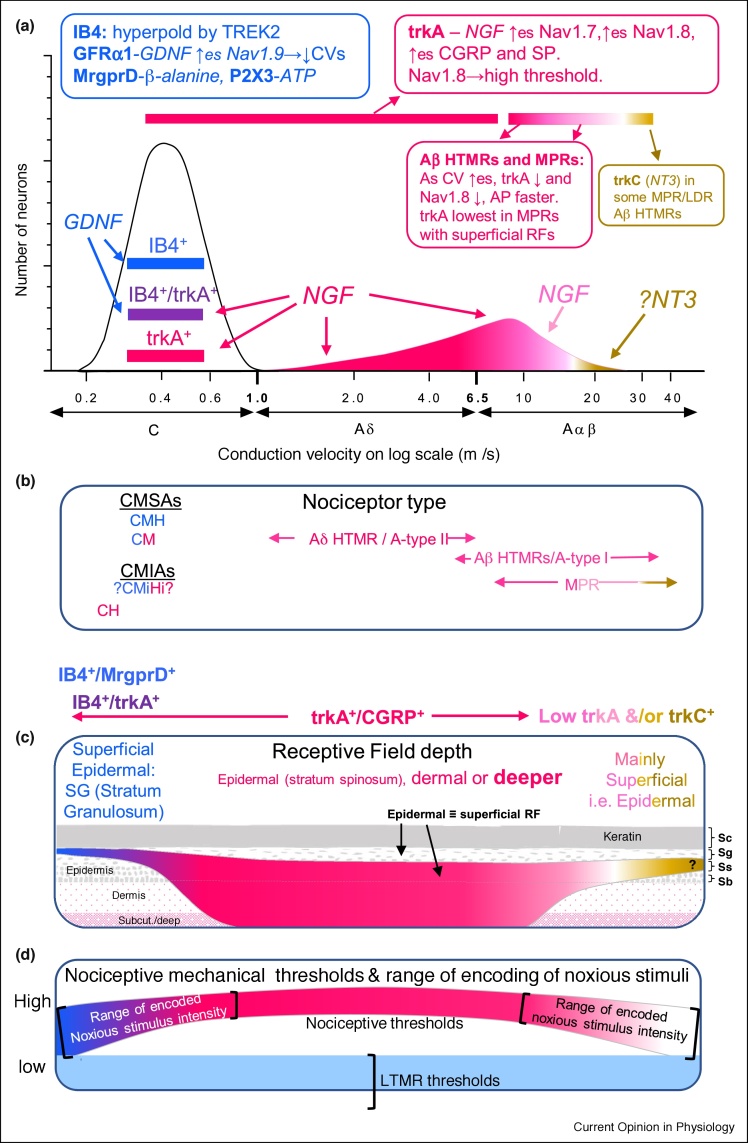
**Relationships between IB4, trkA, CV, mechanical nociceptor types, RF depths, and thresholds.** Diagrammatic illustration relates features across the nociceptor CV range, but below 1 m/s no correlation of the information with CV is intended (section: CVs of C-nociceptor subtypes). The colour code relates to neuronal expressions of IB4^+^/MrgprD^+^ (blue), trkA^+^ (red) and IB4^+^/trkA^+^ (purple) neurons, and mRNA for trkC (orange) see sections: trkA and IB4/MrgprD expressing C-fiber neurons and Chemical phenotypes of A-fiber nociceptors. **(a) Incidence of trkA^+^ or trkC-nociceptors relative to CV for A-fiber neurons.** Summary boxes show some properties of trkA^+^ and IB4^+^/MrgprD^+^ nociceptors: receptors in bolds, ligands for receptors in italics. Arrows: ↑ upregulates, → causes, ↓ decreases. Beside channels are shown properties (lower case) that correlate with the channel expression (immunointensity). Strongly IB4^+^ C-neurons are more hyperpolarised than other C-nociceptors due to their TREK2; they express GFRα1. GDNF acts via GFRα1 to upregulate TREK2 and Nav1.9. Their slower CVs are correlated with higher Nav1.9 expression. The Aδ-HTMR/type II and more slowly conducting Aβ-nociceptors are mostly strongly trkA^+^(red). They show decreasing trkA-expression (pink) with increased CV to no trkA (white) in those with fastest CVs especially the Aβ-MPRs. **(b) Likely nociceptor types in relation to CV >1 m/s are noted, with colour of writing relating to trkA^+^ or IB4^+^ expressions.** CH have slowest CV. CMHs and some CMs are IB4^+^/Mrgprd^+^ (blue). CMiHi neurons may include IB4^+^ and trkA^+^ neurons, but this is not certain (Sections: Unresponsive neurons, CMiHi, silent or unresponsive-neurons), hence the question marks. **(c) RF depths:** In the epidermis, SC is stratum corneum; SG, stratum granulosum; SS, stratum spinosum; SB stratum basale. MrgprD^+^ (thus IB4^+^) fibers terminate in the stratum granulosum (SG), ∼10 μm from the keratin layer in mouse but not in deeper tissues. Their superficial termination sites are a likely/possible contributing factor to their low/variable thresholds **(d)**. The few CGRP^+^ (thus trkA^+^) fibers in epidermis have RFs from stratum spinosum down to subcutaneous and deep. The MPRs have superficial RFs and those with fast CVs express little/no trkA, but some express trkC. **(d) Nociceptive thresholds and the range of mechanical stimulus intensities** that are encoded by firing rates, in relation to RF depths in C and CVs shown in (a). Thresholds of CPMNs and Aβ-MPRs tend to be lower than for most trkA^+^ HTMRs, shown in the middle as higher. Unlike LTMRs, they encode stimulus intensity through the noxious range. Thus on the left are slowly conducting IB4^+^/Mrgprd^+^ CPMNs and on the right, the fastest conducting cutaneous nociceptors, the MPRs. Both have low/no trkA-expression, superficial RFs and generally lower mechanical thresholds than trkA^+^ HTMRs.

### Incidence of CM neurons

The incidence of CM and AM neurons that did not respond to heat or cold are shown in the Pie Charts (deep pink plus purple MPRs, but excluding PMNs) (Figure 3). They can be calculated as for the PMNs in Table 3 legend but using the reciprocals of percentages for PMNs in Table 2a. The percentage of all DRG neurons that are CM units is 38.8%, 1.8% with superficial RFs, 9% with dermal and 28% with deep RFs. However, since the thermal insulation of skin may prevent heat activation of nociceptors with deep dermal and subcutaneous RFs, single cell qPCR [47^••^] could show whether CMs have mRNA expressions that resemble CMH or CMC units. CM units were infrequent in the skin-nerve preparation due to the lack of subcutaneous/deep tissues and possibly loss of some dermal RFs or fibers.

## trkA and IB4/MrgprD expressing C-fiber neurons

The focus here is on two main subtypes IB4^+^ and trkA^+^ particularly in CPMNs. Two-thirds of rat C-fiber neurons show IB4-binding (IB4^+^) but no A-neurons did except for weakly stained Aδ-LTMs [26]. In contrast most C-neurons and most A-nociceptors (especially Aδ and fast Aβ-nociceptors) are trkA^+^ [9]. These patterns may visually suggest that IB4^+^ and trkA^+^ neuronal groups are non-overlapping, and although most rat C-neurons were IB4^+^/trkA^−^ or IB4^−^/trkA^+^, a third of C-neurons were IB4^+^/trkA^+^ (purple Figure 4) with reciprocal immuno-intensities [26] consistent with [47^••^].

### Fiber termination sites

MrgprD^+^ neurons were IB4^+^ and 75% of IB4^+^ neurons express MrgprD in rat DRG [48^••^]. IB4-immunostaining of fibers in skin is not possible due to background staining. However, MrgprD was visualized in DRG neurons and their fibers using genetically encoded axonal tracers [48^••^]. MrgprD^+^ fibers, that is, the fibers of all or most IB4^+^-neurons, project exclusively to epidermis terminating very superficially in the stratum granulosum, within 10 μm of the stratum corneum layer. They do not project to muscle (although see Ref. [47^••^] for a small group of MrgprD neurons), project very little to blood vessels, and to no visceral organs examined [48^••^].

Centrally, IB4^+^ afferent C-fibers terminate in a localised layer between laminae IIo and IIi and between CGRP and PKγC labelling [48^••^]. Transneuronal tracing studies suggested that information from IB4^+^ C-fibers is transmitted to limbic forebrain regions probably contributing to affective responses [49].

CGRP^+^-fibers in epidermis are much more sparse and terminate deeper than MrgprD^+^-fibers, reaching only up to the stratum spinale with much CGRP^+^ innervation in dermis around blood vessels and sweat glands and in deep tissues [48^••^]. However when closely intertwined with MrgprD fibers, CGRP^+^ fibers may also reach the stratum granulosum. In adult rat L4 and L5 DRGs, trkA and CGRP are highly co-localised (90% each way) [50]. Here we therefore assume they label the same neurons/fibers. Thus overall most CGRP^+^/trkA^+^ DRG neurons project to dermis and deep RFs.

In marked contrast to IB4/MrgprD the greatest trkA-immunointensity in mechanical nociceptors was in CMs and A-HTMRs with deep and dermal RFs [9], Figure 2g. This is consistent with retrograde labelling studies showing the highest percentages of rat visceral afferents (75%–99%), then muscle afferents (70%) and lowest of skin afferents (43–51%) were trkA^+^ or CGRP^+^ [51,52].

### Some other receptors or channels in IB4^+^/MrgrpD^+^ C-neurons

**P2X3** is an ion channel activated by ATP. It is expressed in small DRG neurons, preferentially in IB4^+^-C-neurons. Of IB4^+^neurons 68% express P2X3 and of P2X3^+^ neurons, 98% are IB4^+^ [53].

**P2Y1** is an ADP receptor. Of IB4^+^ neurons, 74% expressed functional P2Y1 receptors, and most of those expressed functional P2X3; knockout of P2Y1 caused reduction of noxious thermal sensitivity, both heat and cold, but not mechanical sensitivity in CPMNs [54].

**GFRα1** is expressed by IB4^+^ C-neurons; GDNF acting through GFRα1 maintains expressions of Nav1.9 and TREK2, a K2P-channel, [17,55,56]. Nav1.9 is more highly expressed in C-nociceptive and C-unresponsive neurons than in A-nociceptors [17]. In C-neurons, relative immunointensities of IB4 and Nav1.9 are positively correlated and slower action potential rise times and slower CVs are correlated with higher Nav1.9-immunointensities [17,26].

**TREK2** is selectively expressed in IB4^+^-neurons, and positively correlated with IB4-intensity. Strongly IB4^+^ [26] and strongly TREK2^+^ [55] C-nociceptors are hyperpolarized by ∼10 mV compared with other C-neurons. This hyperpolarization is due to their TREK2 [55,57]. TREK2 limits inflammation-induced spontaneous pain behavior (spontaneous foot-lifting) [55], which is related to C-nociceptor spontaneous firing rates [40]. Thus decreased spontaneous pain behavior may result from the TREK2-induced hyperpolarization decreasing/limiting spontaneous firing [55].

### trkA and CGRP co-expression

trkA is the high affinity receptor for NGF and is expressed by many C-neurons, including C-nociceptors and by about half our unresponsive C-neurons, that are mainly CMiHi/silent neurons (Section: Unresponsive neurons). NGF acting on trkA has many effects. It upregulates the peptides CGRP and substance P, and ion channels including Nav1.7 and Nav1.8. trkA and CGRP are closely co-localised in adult rat DRG neurons [50] (section: Fiber termination sites).

NGF acting on trkA has extensive effects on DRG neurons and is heavily implicated in their sensitization [23,58]. In guinea pig, effects of CFA-induced inflammation, namely decreased action potential duration, increased fiber following frequency and increased percentage of C-neurons showing spontaneous firing, were blocked by a synthetic NGF sequestering protein, tyrosine receptor kinase A Ig2 [59]. In this review however, the main focus is on normal nociceptors, not on extensive changes that occur during inflammation, injury, disease and chronic pain.

## Chemical phenotypes of C-nociceptor subtypes

### CMH-type and CMHC-type CPMNs

Most (about 70%) CMHs are IB4^+^, or Mrgprd^+^ [26,47^••^,60^••^,^61^] in mouse and rat. They also mostly express P2X3 mRNA [47^••^]. In mouse, they did not express trpV1 [43,60^••^,^61^]. In mouse and guinea pig hairy skin CPMNs rarely expressed CGRP [54,60^••^,^62^], but in guinea pig glabrous skin CPMNs were CGRP^+^ [62]. In mouse, expression of mRNAs (single cell qPCR) was seen in most of these CMH-type CPMNs for MrgprD, P2X3 and GFRα2, in many for ASIC2, in most for Nav1.9 and Nav1.8, but only in some for Nav1.7 [47^••^].

Primate CMHs responded quickly (**Q**) or slowly (**S**) to heat. All Q-CMHs were β-alanine-sensitive but only 40% of S-CMHs responded and with a weaker response. β-alanine is a ligand for MrgprD, therefore primate CMHs probably express MrgprD [37]. The Bristol rat CMHs also responded promptly to heat, and the 3 MH-CPMNs tested were strongly IB4^+^ Figure 2c and see [26]. The high proportion of C-nociceptors with superficial RFs that were CPMNs (79%, Table 2b) is consistent with rat CMH fibers projecting superficially and being IB4^+^/MrgprD^+^ in rat as in mouse [48^••^]. The Bristol CMHs probably equate both to β-alanine-sensitive primate Q-CMHs and to murine MrgprD^+^/IB4^+^ CMHs.

#### Transduction and CPMNs: a role for keratinocytes?

Strong evidence that keratinocytes may act as sensory transducers was provided in transgenic mice expressing rhodopsin in keratinocytes. Light activation of these keratinocytes caused firing or increased responses to natural stimuli in most C-nociceptors including all CPMNs [63].

A number of candidate molecules could, if released by keratinocytes, activate these neurons [63]. For CPMN-activation, strong candidates include ATP which activates MrgprD^+^ neurons including CPMNs probably by binding to P2X3 receptors [64,65], and β-alanine [66] which in mouse skin acts on MrgprD to increase sensitivity of neurons including CPMNs to noxious mechanical and heat stimuli [60^••^]. This intriguing topic is in its infancy.

### C mechanocold (CMC) units

CMC units were IB4-negative [9,43,61] and Figure 2c. The few tested were CGRP^+^ or trkA^+^ [9,61].

### CMs or CHTMs

Half the cutaneous CMs were IB4^+^ in mouse [43]. In rat, half the dermal CMs were IB4^+^ (but not strongly, Figure 2c) [26]. Guinea pig CMs with superficial RFs were CGRP^−^ and those with dermal RFs were CGRP^+^ [62] while >60% of rat dermal and deep CMs were trkA^+^ [9]. These findings are consistent with trkA and CGRP being co-localised in adult rat DRG neurons [50] and with trkA^+^ and IB4^+^/MrgprD^+^ being expressed by different C-neurons or having reciprocally related staining intensities [26].

### CMiHi, silent or unresponsive-neurons

Rat unresponsive C-fiber neurons [26] that were fully tested (Section: Unresponsive neurons) with noxious mechanical, heat and cold stimuli were likely mostly the equivalent of primate C-MIAs [36] and silent C-nociceptors. Consistent with these being very high threshold or silent nociceptors, their properties including immunoreactivity for Nav1.9, Nav1.8, IB4-binding and trkA were indistinguishable from those of C-nociceptors, having the full range of IB4 and trkA-immunointensities seen in C-nociceptors [9,17,26,67]. They also have long duration action potentials and after-hyperpolarisations typical of nociceptors in rat and guinea pig [46,68].

In rat, ∼60% of unresponsive C-neurons tested were strongly IB4^+^, and 40% were IB4-negative or weakly IB4^+^, with no intermediate immunointensities which suggests two separate groups Figure 2c and [26]. This is supported by trkA-immunostaining being reciprocally related (linear regression) to the IB4-intensities on the same neurons (Figure 2d). This suggested two groups of C-unresponsive neurons, one strongly **IB4^+^**/trkA^−^ and one IB4^−^/**trkA^+^**. Because most C-unresponsive neurons were CMiHi/C-silent nociceptors (Section: Unresponsive neurons), this pattern probably relates to CMiHis.

### Which C-neuron type/s are IB4^+^?

The present finding that most CPMNs have superficial RFs accords with most of these being IB4^+^/Mrgprd^+^ with their fibers terminating very superficially (Section: Fiber termination sites). The 50% of cutaneous CMs reported to be IB4^+^ (Section: CMs or CHTMs) could also terminate superficially [48^••^] and may account for, or contribute to, the present analysis showing 20% of C-nociceptors with superficial RFs to be CMs. The >50% of our C-unresponsive neurons that were IB4^+^ probably include IB4^+^ CMiHi-neurons, because most C-unresponsive neurons were probably CMiHis (Sections: Unresponsive neurons, CMiHi, silent or unresponsive-neurons).

## A-fiber nociceptors

### A-fiber PMNs

Also see Section: PMNs as a percentage of all DRG neurons. We found no dermal or deep A-PMNs. The percentage of A-nociceptors with superficial RFs that were PMNs diminished from 13.6% for Aδ-nociceptors, to 3.1% for Aαβ-nociceptors (Figure 2a, Table 2b, Section: Incidences at different receptive field (RF) depths). Note, however, that our ∼47 to 49℃ heat stimulus may not have activated A-nociceptors with very high heat thresholds (Section: Thermal stimuli).

### Subtypes of A-fiber nociceptors

A-fiber nociceptors have Aδ-fibers or Aβ-fibers. While Aδ-nociceptors are widely accepted, the existence of Aβ-nociceptors is still not universally accepted despite studies over 50 years, showing that 20–65% of A-nociceptors in species from mouse to monkey have Aβ-fibers [10^••^], see Figure 1a. Despite this evidence, the lack of human data may have contributed to Aβ-nociceptors remaining unrecognised or classed as Aδ. Recently, however, a microneurography study [6^••^] in humans reported Aβ-nociceptors with similar CVs to Aαβ-LTMRs but with higher thresholds. Stimulation of these but not of Aαβ-LTMRs caused pricking, sharp, painful sensations [6^••^], as predicted [5,36].

In the earliest reports by Burgess and Perl [12^••^], cat cutaneous A-nociceptive fibers were subdivided into:

a) **High threshold**, mainly Aδ-HTMRs, probably equivalent to the primate **type II receptors** (below), with high mechanical thresholds.
b) **Intermediate threshold,** with Aδ-fibers and Aβ-fibers and
c) **Moderate pressure receptors (MPRs)**. See Section: Moderate pressure receptors.

#### Types I and II A-nociceptor heat responses

A different classification of monkey cutaneous mechanical nocioceptors was made in terms of their heat responses [5]. The type I heat response was a high threshold (>53℃), long latency and late peak maximum response. This was in Aδ-nociceptors and Aβ-nociceptors with low mechanical thresholds. These are mainly Aβ-nociceptors including MPRs. The type II heat response, with lower heat threshold (median 48℃), short latency and earlier peak response to 53℃ heat stimulus was in Aδ-fiber nociceptors with higher mechanical thresholds, that were thought to cause first pain sensation to noxious heat [5,36]. These appear equivalent to the high threshold Aδ-HTMRs.

#### Moderate pressure receptors (Figure 2)

Despite having low (moderate pressure) thresholds for nociceptors, MPRs were originally described as nociceptors, that encoded stimulus intensity through the noxious range [12^••^]. Their strongest response was to a clearly noxious stimulus which was therefore their ‘adequate stimulus’. They are therefore classed in this review as nociceptors. Recent microneurographic stimulation in humans of Aβ-nociceptors, that probably include MPRs, caused pricking pain [6^••^].

A subgroup of A-nociceptors in mice, many of which had Aβ-fibers, had relatively low mechanical thresholds and encoded intensity through the noxious range; the authors suggested these were MPRs and called them LDRs (Large Dynamic Range) neurons [8]. Unlike the classically described mainly lamina I dorsal horn projections of Aδ-nociceptors their central projection sites were throughout laminae I–V [7,8]. Thus their central termination sites in mouse differ from those of other A-fiber nociceptors.

### Are field receptors Aβ-nociceptors or Aβ-MPRs?

It was suggested [61] that Aαβ-field-LTMRs have properties similar to those of Aβ-nociceptors; indeed some of their units may be Aβ-nociceptors as they encoded mechanical stimulus intensity through the noxious range [61] (Supplement 2 Figure 1). We make no comment about the identity of the circumferential neurons. However, we do not agree that Aαβ-Field-LTMRs and Aβ-nociceptors are the same, because Aβ-nociceptors were clearly distinguished from Aαβ-Field-LTMRs in cat [3,10^••^,^11^], in human where Aβ-nociceptors had thresholds higher than, and not overlapping with, those of Aαβ-Field-LTMRs [60^••^], and in rat (Bristol Data) where action potential durations and after hyperpolarization durations in Aβ-MPRs were much longer (*P* < 0.01 and *P* < 0.0001 respectively) than those in Aαβ-Field-LTMRs [69] with little overlap (Supplement 2 Figure 1) and more typical of nociceptors [46].

### Aβ-nociceptors and MPRs in the Bristol data

In the Bristol data, Aβ-nociceptors were defined as MPRs if they responded to moderate pressure, but responded better to clearly noxious stimuli such as fine pinch or needle pressure (Section: Moderate pressure receptors), following the original descriptions [12^••^]. In our data, most MPRs had Aβ-fibers (Figure 2e), and all had superficial RFs. Approximately 15% of Aδ-nociceptors and 50% of Aβ-nociceptors with superficial RFs were MPRs (Figure 2f), consistent with the original report noting that MPRs and HTMRs ‘were equally common’ [12^••^]. The percentage of A-nociceptors with superficial RFs that were MPRs increased with CV to become the dominant type at CVs >12 m/s (Figure 2f).

### Chemical phenotypes of A-fiber nociceptors

Of Aδ-nociceptors >90% were trkA^+^, all Aβ-nociceptors with CVs <11 m/s were trkA^+^, but no Aβ-nociceptors with CVs > 14 m/s were trkA^+^ [9]. That is, as Aβ-nociceptor CVs increased above ∼11 to 12 m/s, so the median trkA-immunointensity decreased; the fastest conducting including MPRs (Figure 2g) were trkA-negative [9,26]. The median trkA-immunointensities were even higher in Aδ-nociceptors and in the Aβ-nociceptors with slower CVs, than they were in C-nociceptors [9]. Thus trkA expression was very high in A-fiber nociceptors with CVs <12 m/s.

Nav1.8-immunoreactivities and trkA-immunoreactivities were correlated in Aδ-nociceptors and Aβ-nociceptors [9], consistent with their Nav1.8 being upregulated by trkA [70]. In Aβ-nociceptors (not Aδ- or C-nociceptors), shorter duration action potential durations were associated with both lower trkA-expression and lower Nav1.8-expression [9]. The high threshold, that is, depolarized voltage activation threshold [70], of Nav1.8 may contribute to higher mechanical thresholds of more slowly conducting A-HTMRs/type II A-nociceptors, while its slow kinetics may broaden action potentials in A-nociceptors.

Nav1.9 was expressed in 60% Aδ-nociceptors and only ∼30% of Aβ-nociceptors [17,26].

A recent single cell qPCR study of mouse DRG neurons shows that A-HTMRs express mRNA for CGRP and trkA (consistent with their colocalisation [50]), as well as ASIC3 and P2Y2; MPRs (which they call LDRs for Large Dynamic Range), expressed either trkA and CGRP, or trkC and Asic3 [47^••^]. Both A-HTMRs and MPRs expressed mRNA for heavy neurofilament (NFH).

### CV and trkA-expression in Aβ-nociceptors are related to RF depth

MPRs with superficial RFs had the fastest CVs and lowest trkA expressions, non-MPRs with superficial RFs had intermediate CVs and higher trkA-immunostaining, while Aβ-nociceptors with dermal and deep RFs had the slowest CVs and highest trkA-expressions (Figure 2g replotted from [9]).

In Aβ-nociceptors, the change from broad action potentials and slower CVs, to narrower action potentials and faster CVs coupled with more superficial RFs, was gradual [9]. The gradual change in trkA expression with increasing CVs is illustrated diagrammatically in Figure 4a. In the Aδ-HTMR/type II range, strong trkA expression (red) begins to decrease from the low Aβ-CV range (strong pink), and then diminishes further towards no trkA (white) in faster conducting Aβ-nociceptors/MPRs/type I nociceptors, with MPRs with superficial RFs (Figures 2g and 4c) being trkA negative and having lower (moderate pressure) thresholds but encoding stimulus intensity through the noxious range (Figure 4d).

## Discussion and conclusions

These calculation are only as useful as the starting data allow. Here the known problems and limitations have been explained and, where possible, adjustments made. The percentages of PMNs/MHs calculated for the Ca^++^ imaging whole DRG approach and for a variety of electrophysiology approaches replicated the published percentages well, despite large apparent initial differences between them. This is encouraging both for such a mathematical approach and for the validity of the experimental techniques used. However, for useful comparisons between differing techniques, the starting total (100%) of fibers or neurons being examined (e.g. see Table 4) needs to be clear.

### Summary pie charts

Pie charts for the whole DRG, and for C, Aδ and Aαβ are proportioned according to their contributions to the total DRG neuron population in rat L5/L5 DRGs (Figure 3). They illustrate calculated contributions of the different main types of nociceptor to the total DRG neuron population. CPMNs (9.8%) make the dominant contribution to the total PMN population (10.9%) of DRG neurons. MPRs contribute a much smaller proportion of the total (1.6%). The Aδ-neuron contribution overall is very small relative to Aαβ-neurons (Section: Possible bias without adjustment).

### CPMNs

The high percentage of C-nociceptors with superficial RFs that are CPMNs is interesting in view of their possible role in transduction of chemical changes/signalling in the superficial stratum granulosum into trains of impulses (Sections: Fiber termination sites, Transduction and CPMNs: a role for keratinocytes?). Their mechanical thresholds and/or heat thresholds are variable, starting relatively low for nociceptors. Might these lower thresholds reflect their very superficial RFs, their ion channel and receptor complement and/or be dependent on chemicals released by keratinocytes acting act on receptors such as P2X3, P2Y1 and MrgprD expressed on these C-neurons (Section: Transduction and CPMNs: a role for keratinocytes?)?

The importance of the MH-type CPMNs is not reflected by their small 6% contribution to the whole DRG. CPMNs and some CMs are IB4^+^/MrgprD^+^. The IB4^+^/MrgprD^+^ CPMs have terminations in superficial epidermis over the skin. That they make up 80% of C-nociceptors with superficial RFs suggests that their response to external noxious stimuli is very important for protection and integrity of the skin. Pathway tracing indicates that activity in these IB4^+^/MrgprD^+^ neurons can activate limbic forebrain regions and may therefore evoke emotional responses (Section: Fiber termination sites). To speculate, these neurons may be protective, altering affect and changing behaviour and/or contributing to aversive responses to damaging stimuli and dangerous situations. These central pathway connections may also suggest an important contribution to acute or chronic pain-related suffering.

### MPRs and their possible importance

In contrast to C-nociceptors, most A-fiber nociceptors have superficial RFs. Although MPRs are a small population in the DRG (1.6% of the total, Figure 3), they dominate the cutaneous Aβ-nociceptors with the fastest CVs, and with superficial RFs. Despite their small number, in cat MPRs had large RFs with multiple points of ‘heightened sensitivity’ similar to other Aβ-nociceptors with superficial RFs but larger than Aδ-HTMRs [12^••^].

To speculate, their low firing rate to moderate pressure could gain attention well before damage is done, providing an early warning signal [7] (e.g. a small stone in a shoe) that increases with greater or more localised (sharper) pressure or with repeated/continuous pressure thus preparing the body gradually for action allowing time or CNS, muscle groups, cardiovascular system and whole body physiology to prepare for an appropriate response. These MPRs/type I Aβ-nociceptors with the fastest CVs and superficial RFs (Figure 4a) seem ideal for providing gradual increasing recognition of discomfort to initiation of a rapid withdrawal response. This encoding with a sliding scale of firing from low (moderate pressure) to high (damaging noxious stimuli) appears preferable to an information gap between very low (LTMR) thesholds and very high HTMR thresholds.

The precise localisation of a noxious stimulus needs to be signalled, for initiating and co-ordinating a fast withdrawal response. Interestingly, electrical stimulation of Aβ-nociceptors in humans resulted in sharp or pinprick sensations [6^••^] suggesting that precise localisation is signalled to the CNS.

**Little is known about MPRs**. Questions about these neurons include a) in the same MPR fibers, do low firing rates produce a sensation of moderate pressure in human subjects, and high rates cause a sharp sensation? b) do mechanical thresholds of MPRs decrease with inflammation, disease or injury? c) If so, could MPRs contribute to hyperalgesia and/or allodynia? In relation to RFs of Aβ-HTMRs and MPRs, d) where do their fibers terminate in the epidermis? e) in terms of fiber guidance and local influences, what causes these neurons to have superficial RFs? In relation to why MPRs have lower thresholds compared to other Aβ-HTMRs: f) Could their superficial RFs contribute to their lower thresholds; g) could their expression of trkC contribute to their lower thresholds? h) how do they retain their ability to encode for intensity of mechanical stimuli into the noxious range?

### Patterns of mechanical nociceptor properties

Figure 4 is intended as a stimulus to discussion, rather than a textbook diagram. It highlights relationships in normal rats, between some fundamental properties of mechanical nociceptors, including CV, RF depths, trophic factor receptors and thresholds, for which evidence is supplied in the text, in order to question the extent of causality in these relationships. In rat C-neurons, a third were IB4+/trkA− (blue bar), a third were IB4^−^/trkA^+^ (red bar) and a third expressed both, with reciprocal immunointensities (purple bar) [26] see Figure 4. The horizontal bars are used because of the lack of consistent CV difference between these groups other than slowly conducting CH neurons in mouse.

#### IB4^+^/Mrgprd^+^ C-neurons

A) The IB4^+^/MrgprD^+^ C-neurons (blue bar) include most CPMNs, some CMs and possibly some CMiHi (Sections: Unresponsive neurons and 7.5). Both CPMN RFs and IB4^+^/MrgprD^+^ fibers terminate in superficial epidermis (Figure 2a-b, 4c).

#### trkA^+^ neurons

These are presumably also CGRP^+^ (Sections: Fiber termination sites, 6.3, CMiHi, silent or unresponsive-neurons). About two thirds of C-nociceptors (red and purple bars, Figure 4a) (and possibly some CMiHi neurons), express trkA (red) as do Aδ-neurons and slower conducting Aβ-neurons [9]. These are influenced by NGF. Relatively to MrgprD, few have RFs in the epidermis, and many more have RFs in dermal or deeper tissues (Figure 4). They include typical high mechanical threshold nociceptors perhaps because of their deeper RFs and/or their high trkA (red, Figure 4) and combined effects of their ion channel complement including high Nav1.8 expressions. Compared with LTMRs in the same CV groups, they have the typically long action potential and afterhyperpolarisation durations of nociceptors [71].

#### Aβ-nociceptors/MPRs

To the right side of the Aβ-population, the trkA-expression is strong (red). However, as CV increases, the trkA-expression decreases to weak/negative (fading to pink and then white, with some MPRs expressing trkC mRNA in orange Figure 4a), and more of the RFs are superficial (Figure 4c). With increasing CV, the incidence of the lower threshold MPRs increases (Figure 4), and with their lower trkA expresions, decreases the nociceptor phenotype of long action potential durations. It was not clear which trophic factors influence the fastest conducting, trkA-negative MPRs but recent qPCR shows that some MPRs (called LDRs) have trkC mRNA [47^••^], suggesting that some MPRs may be influenced by NT3.

#### The two extremes

The CPMNs and Aβ-nociceptors (including MPRs) are contrasting groups of nociceptors in terms of CV (some of the slowest-plus the fastest-conducting cutaneous nociceptors) and action potential durations, broadest (IB4^+^ C-neurons) and narrowest (Aβ-MPRs).

Despite this, there are similarities. Both MrgprD^+^/IB4^+^ CPMNs and MPRs showed low or no trkA/CGRP-expression, presumably limiting the influence of NGF. This differs from most other A-fiber and C-fiber nociceptors. MPRs and many CPMs have lower thresholds and more superficial RFs than many other clearly NGF^+^ nociceptors (Figure 4). The IB4^+^/MrgprD^+^ CPMNs have fibers terminating in very superficial epidermis. In contrast few trkA^+^ nociceptors have fibers penetrating the epidermis and usually these reach only the stratum spinale, deeper than MrgprD^+^/IB4^+^ fibers. The majority of trkA^+^ nociceptors have dermal and deep RFs.

Although at the opposite ends of the CV range, their superficial RFs means that CPMNs and MPRs are both important for responding to external potentially noxious stimuli. The CPMNs (Table 5) respond more slowly, likely causing emotional responses but not signalling precise information about localisation. The Aβ-nociceptors/MPRs may begin signalling with moderate pressure causing initial awareness, but when the stimulus increases into the noxious range, their fast afferent CVs and highly localised (pricking) sensation would enable accurate and rapid withdrawal from a potentially damaging mechanical insult.

**Table 5:** Abbreviations.

CAP, compound action potential
CH, C-MIA that responds to noxious heat
C-HTMR, C-fiber high threshold mechanoreceptor
CLTM: C low threshold mechanoreceptors
CPMN, C-fiber polymodal nociceptor
CMC, C-fiber nociceptor responding to noxious mechanical and noxious cold
CMH, C-fiber nociceptors that respond to noxious mechanical and noxious heat stimuli
CM or CHTM, C mechanonociceptor
C-MIA, C mechano-insensitive neuron that normally does not respond to noxious mechanical stimuli
CMiHi, C-fiber mechano-insensitive, heat-insensitive neuron
C-neuron, C-fiber neuron
Cunr, C-fiber unresponsive unit
CV, conduction velocity
DRG, dorsal root ganglion
Em, resting membrane potential
GCaMP, Genetically-encoded Calcium Indicator
GDNF, glial cell derived neurotrophic factor
HTMR, high threshold mechanoreceptor
LL, large light neuron
LTMR, low threshold mechanoreceptor
MC, nociceptor responding to noxious mechanical and noxious cold stimuli
MH, nociceptor responding to noxious mechanical and noxious heat stimuli, MHC, nociceptor responding to noxious mechanical, noxious heat and noxious cold stimuli
MPR, moderate pressure receptor
Mrgprd, Mas-related G protein-coupled receptor D, a subclass of Mrgprs that co-localises with IB4-binding in DRG neurons
Nav, voltage-gated sodium channel
NF, neurofilament
NF200, the large (200kD) NF-subunit
NGF, nerve growth factor
SD, small dark neurons
PMN, polymodal nociceptor
RF, receptive field
trkA, high infinity rceptor for NGF
TF, trophic factor
unr or unresp, unresponsive

#### Summary

Literature review combined with reanalysis of a database of intracellularly recorded neurons to determine nociceptor subtype incidence amongst all rat lumbar DRG neurons, focussing especially on C-polymodal nociceptors (CPMNs) and Aβ-moderate pressure receptors (MPRs), found the following.

1 About 11% of **all nociceptive DRG neurons** were calculated to be polymodal nociceptors (PMNs) similar to reports in a Ca^++^-imaging study [1^••^].
2 Up to 79% of **cutaneous C-nociceptors** were CPMNs, while up to 58% were CMHs, are consistent with electrophysiological studies on cutaneous nerve fibers (65–85% cutaneous C-nociceptors are CPMNs).
3 Of C-nociceptors, 18%, 25% and 58% had superficial, dermal and deep receptive fields (RFs), and 80%, 25% and 0% of C-nociceptors with superficial, dermal and deep RFs respectively were CPMNs. Thus CPMN RFs tend to be very superficial, while most C-nociceptors have deep or dermal RFs.
4 Aβ-nociceptors made up 74% of A-fiber nociceptors. Although moderate pressure receptors (MPRs) were only 6% of Aβ-nociceptors, they were 50% of Aβ-nociceptors with superficial RFs.
5 Aβ-MPRs (the fastest conducting nociceptors) have low trkA-expression, low nociceptive mechanical thresholds, and a high proportion of superficial RFs. IB4^+^/MrgprD^+^ C-fiber PMNs also have low trkA expression and superficial RFs.
6 Other nociceptor types that express trkA tend to have have deeper RFs and higher thresholds to externally applied stimuli.

## Funding

The recordings and immunocytochemistry were carried out in the University of Bristol, UK, funded by The Wellcome Trust (Grant number 06842, 082508 and 072030), and the British Biological Science Research Council (BBSRC) (Grant number 7/S14627). Papers on this research are published. This analysis of the data is novel.

## Conflict of interest statement

Nothing declared.

## References and recommended reading

Papers of particular interest, mostly published within the period of review, have been highlighted as:

• of special interest
•• of outstanding interest
