# Supplementary material for: Nociceptor subtypes and their incidence in rat lumbar dorsal root ganglia (DRGs): focussing on C-polymodal nociceptors, Aβ-nociceptors, moderate pressure receptors and their receptive field depths
Source: Curr Opin Physiol. 2019 Oct;11:125–46. doi: 10.1016/j.cophys.2019.10.005 (PMC6959836; doi:10.1016/j.cophys.2019.10.005)
Supplement: Supplementary file 1 [file mmc1.docx]

Supplementary Methods for:

*Nociceptor subtypes and their incidence in rat lumbar dorsal root ganglia (DRGs): focussing on C-polymodal nociceptors, Aβ-nociceptors, moderate pressure receptors and their receptive field depths by* Lawson, S.N., Fang,X. and Djouhri, L. in Current Opinions in Physiology.

Figures, graphs and Tables (except Fig. 1C-D, and Fig. 2C and F) are based on re-analysis of the Bristol Database of >1000 neurons recorded in normal rat L4/L5 DRG neurons by Dr. Laiche Djouhri and Dr. Xin Fang in Bristol. All recordings were on young adult female rats, under deep anaesthesia [1,2]. Most of these neurons were included in previous publications, but the analyses of relative incidence of the different sensory subtypes reported here are novel. Some aspects of previously published work are referred to, and in each case specific reference is made to the paper in which the data are published.

1. **ANIMAL PREPARATION**

All Experimental procedures complied throughout with Home Office guidelines (United Kingdom). Rats were young female adult Wistar rats (~7 weeks of age, 150–182g median 186g) were deeply anesthetized initially with sodium pentobarbitone (70–80 mg/kg, i.p.). They were maintained deeply anaesthised (areflexic) with supplementary doses (10 mg/kg) throughout the experiment.

At the end of the experiment the rat was terminally anaesthetized.

The fur on the left foot and leg was clipped short. The left external jugular vein and carotid artery were cannulated to allowed supplementary anaesthetic at regular intervals and monitoring of blood pressure. Animals were intubated for artificial ventilation. End-tidal CO_2_ was monitored throughout. After laminectomy, a long spinal clamp was used to fix the vertebral column, reducing movement and vibration of the DRGs. The left L3–L6 DRGs were exposed, and a pool was constructed using silicone dental impression paste Xantopren VL Plus (Heraeus Kulzer, Hanau, Germany). Bone was excavated from below the DRG or DRGs to be recorded, and a silver platform was inserted under the DRG and raised very slightly while observing with a binocular microscope to ensure that the DRG capillaries continued to perfuse the DRG and did not blanch. This platform raised the DRG slightly from the underlying tissues, especially bone, to isolate the DRG from cardiac and respiratory movements and also from manipulation of tissues with hand-help stimulators.

The pool was filled with liquid paraffin before recording began. The temperature close to the DRGs was monitored and maintained between 28.5 and 32°C throughout. The spinal cord dura was cut open and the dorsal root for the DRG to be recorded was cut close to its spinal cord entry just before recording from that DRG. The dorsal root was placed over a pair of platinum stimulating electrodes with maximum distance between stimulating electrode and DRG.

**1.1 Leg position for recording**

The left leg was extended before recording. The foot was plantar surface upwards. It was held in position by gluing the dorsal surface of the foot downward to a stainless steel platform beneath, using Loctite super glue (Henkel, Dublin, UK). This held the foot rigidly in place. These measures increased recording stability.

**1.2 Muscle relaxant**

Just before recording, and to further improve recording stability, a muscle relaxant, pancuronium bromide (0.6 mg/kg, i.v.) accompanied by anesthetic (10 mg/kg, i.v.) were administered and the rat was artificially ventilated. Regularly (at ~1 hr intervals) muscle relaxant and anesthetic the same doses of were given together throughout recording (usually ~3 h).

1. **INTRACELLULAR RECORDING**

A sharp glass microelectrode was advanced in 1µm steps until intracellular voltage recordings from DRG neuronal somata. Neuron penetration was often aided by a brief high-frequency pulse of current, although such pulses were used minimally in dye-filled electrodes (see below), especially those containing ethidium bromide, to avoid leakage of dye [3]. When a resting membrane potential (Em) was obtained, electrical stimulation was applied to the dorsal root with single rectangular pulses (duration, 0.03 ms for A-fiber neurons or 0.3 ms for C-fiber neurons). The stimulation voltage was 1–2 times the threshold for evoking a somatic AP. These APs were recorded online with a CED 1401 plus interface (Cambridge Electronic Design, Cambridge, UK) and the SIGAV program (Cambridge Electronic Design).

**2.1 Electrode contents**

Some recordings were made with 3M KCl filled electrodes, others were filled with a fluorescent dye. The fluorescent dyes were: 50 mg/ml Lucifer yellow CH (Sigma, St. Louis, MO) in 0.1 M LiCl, 6 mg/ml ethidium bromide (Sigma) in 1 M KCl, 3% cascade blue (Invitrogen, Eugene, OR) in 0.1 M LiCl. The >1000 neurons included in this analysis were recorded using the following types of electrodes, KCl 22%, CB 10%, EB 22% and LY 46%. This means that 44% were recorded in KCl electrodes, and the rest with LiCl electrodes. In the C-nociceptor population these proportions were 79% LiCl (including 58% LY and 10% CB) and 37% KCl (16% 3M KCl and 15% EB). These proportions reflect the aim of these experiments. Most were to dye-inject neurons for immunocytochemistry. This was limited by it being possible only to confidently find a small number (usually 4-6) dye-injected neurons per DRG per experiment, coupled with our aim being to dye-inject C-fiber neurons and all nociceptors, such that some Aαβ-LTMRs would be rejected early in the experiment when dye-filled electrodes were being used. Full details on issues with finding dye-filled neuronal sections are available [3].

In the analysis in the article, all neurons regardless of electrode content were included on the basis that the higher electrode resistances with LiCl as the electrolyte would not the ability to record action potentials conducted along the dorsal root, evoked by dorsal root stimulation, or along the peripheral nerve, due to successful stimulation of sensory receptive field from reaching the neuronal soma. For dye-injection, LY-electrodes were usually used first, being the most reliable dye in our experience. Note that some neurons recorded with dye filled electrodes may not have had dye ejected, and that in any case, all the sensory receptor type evaluation was prior to dye ejection, so was unlikely to have any effect on the neuronal soma during sensory testing.

**2.2 Conduction velocity (CV).**

CV was determined by division of the conduction

distance by the latency. The conduction distance was between the cathode of the dorsal root stimulating electrode pair and the neuron in the DRG being recorded and was median 8.3mm with 25% and 75% percentiles of 6.8 and 10mm. The latency

was from the stimulus artifact to the onset of the evoked somatic action potential as previously described and the limits of accuracy discussed, for details of CV calculations see [4]. Utilization time was not taken into account. The dorsal root fiber CV of each neuron was classified as C <1m/s, Aδ >1-6.5m/s and Aαβ ≥6.5m/s, determined by distribution histograms of CVs of identified neurons and confirmed by compound action potentials on the same dorsal roots in rats of the same sex and weight as the experimental animals in this paper (Fang et al., 2002).

**2.3 Spontaneous activity.**

Before the receptive field was stimulated (see below), the presence of any spontaneous/ongoing activities was recorded usually for 1–2 min. Even 1 non-evoked AP during this time would be classed as spontaneous firing. However, no calculations were made in this analysis on neurons relative to any spontaneous firing.

1. **SENSORY RECEPTIVE PROPERTIES AND RECEPTIVE FIELD (RF) DEPTH**

A search for the receptive properties and receptive field depth in the tissue was first made with hand-held stimulators.

**3.1 Access to hindlimb for stimulation:**

Of the L4 and L5 DRG receptive field regions, the following were completely accessible for mechanical and thermal stimulation: the hairy skin of the lateral thigh, leg, heel, foot and toes (all free and movable) plus all the (highly innervated) glabrous skin. Most less accessible regions of the leg (thigh: ventral/anterior and medial surfaces, and medial side of the foot) are innervated by L3 and are thus not relevant here [5].

**3.2 Low threshold mechanoreceptors (LTMRs)**

Non-noxious stimuli were tested first: brush, light touch, moderate pressure, tapping, vibration. Neurons responding to non-noxious mechanical stimuli were classified as low threshold mechanoreceptors (LTMs). Slowly conducting LTMs included C-fiber LTM units (C LTMs) or Aδ down hair (D hair) units. Aαβ-LTMs were subdivided as described [2,3,6,7] but here they were included as one Aαβ-LTM group except for those that were defined as Field type 1 and Field type 2 receptors, see Supplementary Fig. 1.

**3.3 High threshold mechanoreceptors (HRMRs)**

If there was no response to non-noxious stimuli, noxious mechanical stimuli were used to search for the RF. Moderate pressure was applied, often with rounded stainless steel rod. A response to moderate pressure but a greater response to more overtly noxious stimuli was indicative of a moderate pressure receptor (MPR). A superficial (probably epidermal) RF was indicated if the unit fired in response to gentle non-penetrating pressure with a fine needle. A superficial RF would also be activated by pinch of the superficial skin with fine no. 5 forceps, which probably stimulates RFs in the epidermis or at the deepest possibly the epidermal/dermal junction. If none of these caused activation, squeeze/pinch across a fold of skin including dermis was tried. If this caused activation, the RF was likely dermal. If none of the preceding activated the neuron, squeezing with flat or toothed forceps across a muscle, limb or foot, including laterally across the foot, would be tried. Activation here indicated a deep RF (deep fascia, muscle or periosteum).

**3.4 Thermal stimulation**

Thermal stimulation followed: cooling with a brief localized spray of ethyl chloride and/or 50°C water from a 20 syringe with no needle in place was aimed at the mechanical RF or over the whole region, if no RF had been found.

Upper layers of the skin are good thermal insulators. Heat stimuli may not penetrate adequately to the terminals of all dermal units, or of any deep units. It was unusual to obtain a response to noxious thermal stimuli unless the mechanical RF was superficial or dermal (less likely) or if the unit was thermally but not mechanically activated (in which case it is hard to determine the RF depth). Nociceptive units with deep RFs were rarely tested with thermal stimuli because they so rarely responded. In the present analysis of PMN incidence, all units that were tested for heat were included. That is, all units in PMN calculations were tested with noxious mechanical, noxious heat, and many also with noxious cold.

The low percentage of nociceptors with non-superficial RFs that are PMNs is hard to interpret. Without thermally damaging the tissues it is not possible to determine whether nociceptors with non-superficial mechanical RFs are thermally unresponsive, or whether they are thermally responsive but thermally well-insulated thermally by epidermis and dermis. This is discussed more in the article.

- 1. **Unresponsive neurons**

Neurons that responded to none of the above stimuli (mechanical, thermal, non-noxious or noxious) applied over the L4/L5 innervated regions (Section 3.1) were called unresponsive, also see Section:2.3, main paper.

Of all the accessible RFs in the database, none were heat only nociceptors. Unless the C-fiber heat nociceptors (CH) are only present on the dorsal surface of the foot, it seems unlikely that the C-mechano-insensitive neurons were CH units. They were more likely to be C-silent or CMiHi units.

**3.5.1 Possible Underestimation:** If not fully tested with mechanical and heat stimuli, they are not declared unresponsive. The extensive searches and increasing intensities of mechanical stimuli increase the likelihood of loss of the recording before they can be declared fully unresponsive. This group is therefore probably underestimated compared with neurons with identified RFs, but we do not know by how much.

**3.5.2 Inaccessible RFs in Glued region:** The majority of foot and leg regions innervated by L4 had RFs that were easily accessible for stimulation (Section 3.1). The only region not accessible to direct stimulation was the glued down patch (mid-dorsal foot hairy skin surface). This region is innervated mainly by L4, and is an area of about ~10-15% of L4-innervated hairy skin on the hind limb. L5 innervates the lateral side of the foot and toe. The toes were not glued down, and were easy to stimulate and thus L5 RFs would have been accessible to stimulation, by squeezing the lateral toe and laterally across the foot [5]. Importantly all glabrous surfaces were accessible and these are more highly innervated than the hairy skin.

This matters only for determining the percentage of neurons that were classed as unresponsive but that had inaccessible RFs. Below we estimate this.

**3.5.3 Estimate of C-unresponsive neurons with inaccessible RFs**

L5 C-neurons in this study made up ~80% of all L4/L5 neurons. The percentage of L4/L5 unresponsive C-neurons that were L4 (21%) is similar to that of L4/L5 C-nociceptors that were L4 (18.4%). The higher percentage of L4 neurons in unresponsive than nociceptive C-neurons was L4 was 100X(2.6/21)%=12.3%. Thus of C-unresponsive neurons ~12-15% of L4 neurons and a much lower percentage of L5 neurons, may have had inaccessible RFs due to their receptive fields being in glued down skin. Taking 15% as the value for L4 and factoring in that only 20% of C-unresponsive neurons were L4, the total percentage likely of C-unresponsives likely to to have been falsely identified due to unresponsive RFs is about 0.2x0.15x100%=3%.

While this seems low, it may be explained as follows. 1) the small hairy skin area glued down and 2) the higher innervation density of fully accessible glabrous skin than hairy skin suggests that overall the accessible RFs would vastly dominate over the foot and leg L4/L5 neuron innervation (Section 3.1). In addition, some C-nociceptors with RFs beneath the glued surface may have been activated by deep squeezing medio-laterally across the foot, and/or pressure on the plantar surface, including by shearing forces between glued surface and RFs beneath.

LTMRs were not likely to be classed as unresponsive, because they would have been activated by vibration/slight movement even beneath the glue. This is supported by their soma electrophysiology (long action potential and afterhyperpolarization durations) and immunocytochemical properties being similar to those of nociceptors in all CV ranges (see Section:7.4, main paper), making them good candidates for being mostly silent nociceptors/Mechano-Insensitive Afferents (MIAs).

We therefore conclude that the vast majority of our unresponsive C-neurons are equivalent to CMiHi and silent nociceptors.

**Immunocytochemistry of dye-injected neurons**

Two papers give the details of dye injection and immunocytochemistry referred to in this review. The first deals with the problems of dye-injecting neurons and how to avoid issues of dye-leakage causing falsely labelled neurons [3]. The second provides the details of immunocytochemistry used on the dye injected neurons to show IB4-binding and trkA-expression [6].

1. **ESTIMATION OF INCIDENCES OF NEURONAL SUBTYPES**

**Bias**: The method of intracellular recording is unavoidably biased.

**Size:** There is bias towards larger neurons, especially fast-conducting, large, Aαβ-fiber neurons. This is a) because of greater ease of penetrating large neurons and b) because recordings from these are more stable. This stability results partly from the large neuron volume, and partly because sensory properties of LTMs are much easier to identify without destabilizing the recording, being easily stimulated with very gentle mechanical skin stimulation. The harder the RF is to stimulate, the greater the likelihood of losing the recording due to movement. Neurons with deep receptive fields requiring squeezing, across the foot or leg, and unresponsive/silent neurons, that require a full repertoire of tests are most likely to be lost before characterisation is complete. Because of these factors, Aαβ-LTMs tended to dominate the data set. Some of these were therefore rejected during experiments, to avoid them swamping the data set, and in order to focus our studies on nociceptors.

**Adjustment 1:** To determine the extent of this bias, six experiments were carried out by X. Fang, to include all neurons, with no rejections. The ratio of Aβ-nociceptors to Aαβ-LTMRs in these experiments was 21.4%:78.6%. This was extremely close to the percentage of Aβ-neurons that showed inflections on the falling phase (23% [8]), something known to be associated with nociceptors [9]. The present data set had an Aαβ-LTM incidence that was ~36% lower than the incidence in these unbiased experiments, suggesting, very plausibly, that we had rejected ~36% of these during experiments. We therefore multiplied the Aβ-LTM population by 1.364, increasing it to 775, to achieve a ratio of 211:775, equivalent to the unbiased 21.4%:78.6% ratio. This compensated for Aαβ-LTMRs rejected during experiments.

**Adjustment 2:** C-fiber neurons, despite their large numbers, are much harder to record from than other neurons, even than Aδ-neurons. However, the small neuron population in the DRG is made up of the C-fiber neurons, and because this makes up 70% of the rat L5 DRG using unbiased counting methods [10] we adjusted the C-neuron population up to 70% of the total. This required multiplying the recorded C-neurons by 16.07. Again, this seems a plausible correction given how few of these neurons can be recorded during an experiment compared to the larger neurons.

Within the C-fiber neuron population, or within the Aδ-neuron population, we have no reason to suspect bias. We therefore make comparisons within these ranges with no adjustments.

**Aδ-fiber neurons**: Although it is easier to make stable recordings from Aδ- than C-fiber neurons**,** fewer were recorded than the larger neurons. We cannot exclude a possibility that Aδ- may be underestimated relative to Aαβ-neurons in intracellular recording experiments. However, if the proportion Aδ-neurons in the DRG was much greater than we calculate, this would increase the numbers of NF-rich neurons in the Aδ-neuron size range. The lack of a consistent peak in the Aδ-neuron size range (see Fig. 1C) of the NF-rich neuron distribution suggests that the Aδ-population in the DRG may really be small as suggested in Fig. 1D, Fig. 3. The bias may not be great, and we make no compensation because we have no objective method of determining or compensating for any such bias.

1. Fang X, Djouhri L, McMullan S, Berry C, Okuse K, Waxman SG, Lawson SN: **trkA is expressed in nociceptive neurons and influences electrophysiological properties via Nav1.8 expression in rapidly conducting nociceptors**. *J Neurosci* 2005, **25**:4868-4878.

2. Fang X, McMullan S, Lawson SN, Djouhri L: **Electrophysiological differences between nociceptive and non-nociceptive dorsal root ganglion neurones in the rat in vivo**. *J Physiol* 2005, **565**:927-943.

3. Lawson SN, Crepps BA, Perl ER: **Relationship of substance P to afferent characteristics of dorsal root ganglion neurones in guinea-pig**. *J Physiol* 1997, **505 ( Pt 1)**:177-191.

4. Djouhri L, Lawson SN: **Increased conduction velocity of nociceptive primary afferent neurons during unilateral hindlimb inflammation in the anaesthetised guinea-pig**. *Neuroscience* 2001, **102**:669-679.

5. Takahashi Y, Nakajima Y, Sakamoto T: **Dermatome mapping in the rat hindlimb by electrical stimulation of the spinal nerves**. *Neurosci Lett* 1994, **168**:85-88.

6. Fang X, Djouhri L, McMullan S, Berry C, Waxman SG, Okuse K, Lawson SN: **Intense isolectin-B4 binding in rat dorsal root ganglion neurons distinguishes C-fiber nociceptors with broad action potentials and high Nav1.9 expression**. *J Neurosci* 2006, **26**:7281-7292.

7. Djouhri L, Bleazard L, Lawson SN: **Association of somatic action potential shape with sensory receptive properties in guinea-pig dorsal root ganglion neurones**. *J Physiol* 1998, **513 ( Pt 3)**:857-872.

8. Waddell PJ, Lawson SN: **Electrophysiological properties of subpopulations of rat dorsal root ganglion neurons in vitro**. *Neuroscience* 1990, **36**:811-822.

9. Lawson SN: **Phenotype and function of somatic primary afferent nociceptive neurones with C-, Adelta- or Aalpha/beta-fibres**. *Exp Physiol* 2002, **87**:239-244.

10. Tandrup T: **Unbiased estimates of number and size of rat dorsal root ganglion cells in studies of structure and cell survival**. *J Neurocytol* 2004, **33**:173-192.
