# Supplementary material for: Nociceptor subtypes and their incidence in rat lumbar dorsal root ganglia (DRGs): focussing on C-polymodal nociceptors, Aβ-nociceptors, moderate pressure receptors and their receptive field depths
Source: Curr Opin Physiol. 2019 Oct;11:125–46. doi: 10.1016/j.cophys.2019.10.005 (PMC6959836; doi:10.1016/j.cophys.2019.10.005)
Supplement: Supplementary file 2 [file mmc2.pdf]

[62]

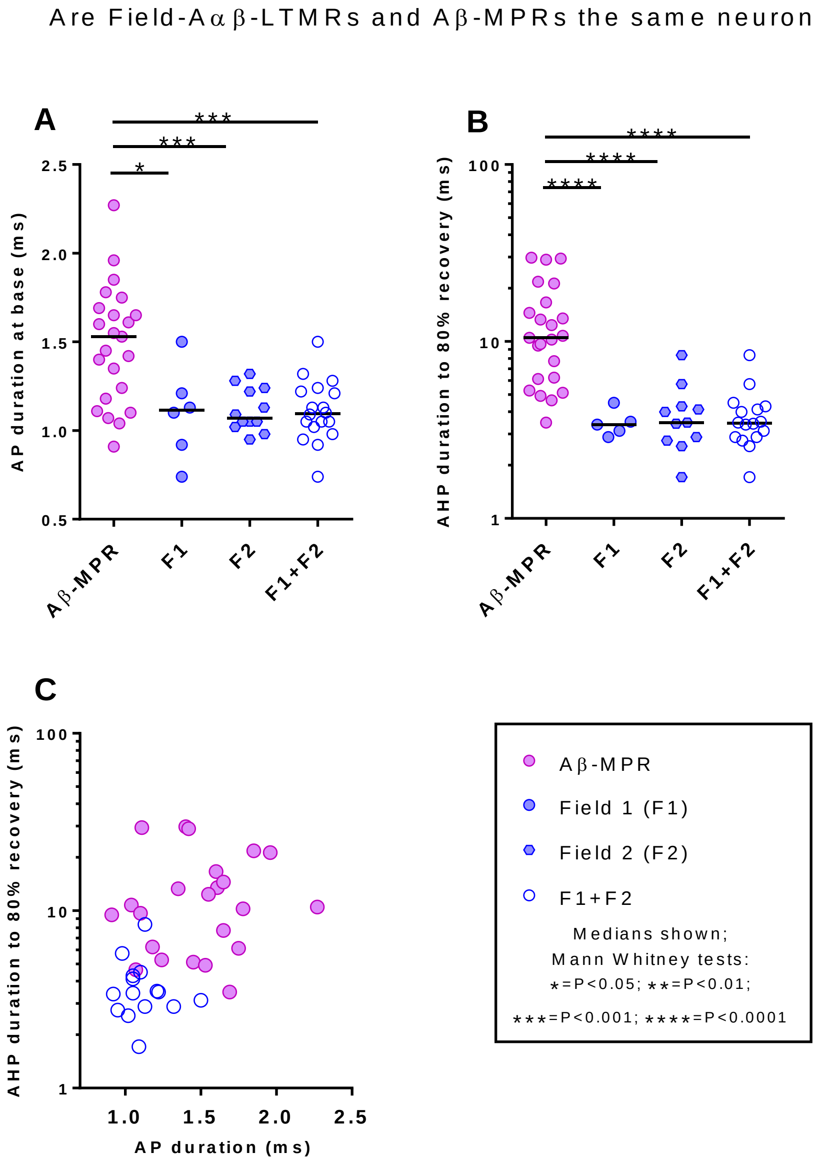

Supplementary Figure 1:

Title: **Are Field Aαβ-low threshold mechanoreceptors (Field-Aαβ-LTMRs) and Aβ-nociceptors that are moderate pressure receptors (Aβ-MPRs) the same group of afferents?**

These are all the units from the Bristol Data Set that were recorded with intracellular electrodes filled with KCl, 1M or 3M, that had a membrane potential of at least -40mV, an overshooting action potential and fitted the criteria for identification of these two groups (see main paper). The records were of dorsal root evoked somatic APs recorded intracellularly in identified Aβ-nociceptors that were MPRs (red/pink) and in Field Aαβ-LTMRs types 1 and 2 (blue), for Methods see [1,2].

A) Action potential (AP) and B) afterhyperpolarisation (AHP) duration to 80% recovery. Differences between all Field and MPR neurons were highly significant in A) and B). C) AP duration versus AHP duration

Continued on next page

Although there is a small overlap in these properties (C), most are in one group or the other. Here we include only those fully identified during the experiment and recorded as Field-type 1 or Field type 2.

The comparison was made against Aβ-MPRs rather than all Aβ-nociceptors, because some properties of Aβ-MPRs show a less extreme nociceptive phenotype that other Aβ-nociceptors [1] and are thus most likely to have properties overlapping with those of any Aαβ-LTMR subgroup. However, their small overlap suggests that even Aβ-MPR and Field-Aαβ-LTMRs are two distinct groups of afferents.

*NB Because of the time taken to distinguish between them and because our focus was mainly on nociceptors, some units were grouped as F/G (Field or G hair) during the experiments. Although many were fully identified during experiments, they were grouped as G hair/Field in most of our publications because we saw no electrophysiological difference between these groups when fully identified.

1. Bai L, Lehnert BP, Liu J, Neubarth NL, Dickendesher TL, Nwe PH, Cassidy C, Woodbury CJ, Ginty DD: **Genetic Identification of an Expansive Mechanoreceptor Sensitive to Skin Stroking.** Cell 2015, 163:1783-1795.

2. Fang X, McMullan S, Lawson SN, Djouhri L**: Electrophysiological differences between nociceptive and non-nociceptive dorsal root ganglion neurones in the rat *in vivo***. J Physiol 2005, 565:927-943.

3. Horch KW, Tuckett RP, Burgess PR: **A key to the classification of cutaneous mechanoreceptors**. J Invest Dermatol 1977, 69:75-82.
